# Immune response to DNA and RNA: structural insights, molecular mechanisms, and therapeutic targeting

**DOI:** 10.1186/s43556-026-00537-x

**Published:** 2026-08-06

**Authors:** Lintao Xia, Yixi Wang, Xiuli Yan, Hui Zhang

**Affiliations:** 1 Institute of Interdisciplinary Integrative Medicine Research, Shanghai University of Traditional Chinese Medicine, Shanghai, China; 2 Department of Minimally Invasive Spine Surgery and Precision Orthopedics, The First Affiliated Hospital of Xinjiang Medical University, Urumqi, China; 3 Yueyang Hospital of Integrated Traditional Chinese and Western Medicine, Shanghai University of Traditional Chinese Medicine, Shanghai, China

**Keywords:** CGAS–STING, RIG-I/MDA5, DNase family, RNase family, Nucleic acid sensing

## Abstract

The recognition of mislocalized DNA and RNA by cGAS–STING, RIG-I/MDA5, the OAS–RNase L axis, and endosomal TLR3/7/8 has emerged as a unifying paradigm linking cancer, autoinflammation, and antiviral immunity. Counterbalancing these sensors is a structurally heterogeneous nuclease repertoire whose distinct substrate specificities, subcellular compartments and pH optima constrain ligand availability in space and time. Disruption of this equilibrium drives disease through two mirror-image mechanisms. In cancer, DNASE1 is inactivated by tumor-derived G-actin, DNASE1L3 is transcriptionally silenced in hepatocellular, colorectal and lung adenocarcinomas, and DNASE2 is upregulated in immunologically “cold” tumors, together permitting neutrophil-extracellular-trap–mediated exclusion of cytotoxic T cells and suppression of cytosolic DNA sensing. In autoimmunity, biallelic loss of DNASE1L3, TREX1, RNase H2, ADAR1 or RNase T2 produces the interferonopathies of systemic lupus and Aicardi–Goutières syndrome. Diagnostically, nuclease-specific cleavage signatures have matured into cell-free DNA fragmentomics validated across 13 cancer types in a 3,021-patient cohort; therapeutically, the field now spans engineered actin-resistant DNASE1/DNASE1L3 biologics, selective TREX1 and ADAR1 inhibitors entering first-in-human evaluation, STING-activating nanomedicines, RNase Fc-fusions such as RSLV-132 in phase 2a lupus, and JAK1/2 inhibition as standard of care in Aicardi–Goutières syndrome. We synthesize this evidence as a two-fate problem: whether an endogenous nucleic acid accumulates at these sensors to drive autoinflammation or is cleared by nucleases before detection is set by the balance between sensor engagement and clearance capacity. The therapeutic corollary acts on the ligand rather than the enzyme, restoring ligand availability where disease is malignant and restoring clearance where disease is self-directed.

## Background

The compartmentalization of DNA to the nucleus and mitochondria and of RNA to its canonical subcellular niches is not incidental cellular housekeeping but a fundamental principle of eukaryotic self–non-self discrimination [1, 2]. When nucleic acids appear outside their designated compartments or in unmodified chemical forms, the innate immune system interprets this mislocalization as a danger signal indicative of infection or breakdown of cellular homeostasis [3]. To counteract such threats, mammals have evolved a diversified surveillance network in which the DNA arm, organized around cyclic GMP-AMP synthase (cGAS)–stimulator of interferon genes (STING) together with the AIM2 inflammasome and Toll-like receptor 9, operates in parallel with the RNA arm, organized around retinoic acid–inducible gene I (RIG-I), melanoma differentiation–associated protein 5 (MDA5), the OAS–RNase L axis, and the endosomal TLR3, TLR7 and TLR8. Historically, the DNases and RNases coupled to these sensors were viewed through a narrow “housekeeping” lens confined to digesting dietary nucleic acids or clearing dying-cell debris [4, 5]. The genomics era, together with major progress in tumor immunology, antiviral immunology and the interferonopathies, has decisively overturned this view: the DNASE1, DNASE1L1, DNASE1L2, DNASE1L3, DNASE2 and DNASE2B enzymes on the DNA arm, and RNase L, RNase H1/H2 and RNase T2 on the RNA arm, are now recognized as dynamic, context-dependent regulators of immune sensing. Phylogenetic analysis dates the diversification of the DNase repertoire to the early-vertebrate radiation approximately 650 million years ago, while the RNase H lineage and OAS–RNase L axis predate the eukaryote–prokaryote split, indicating that nucleic-acid clearance is an ancient and evolutionarily conserved layer of host defense [6, 7].

Progress over the past three years has redefined what is clinically actionable in this field, and the density and directional consistency of these advances motivate the present review. Structurally, high-resolution work has resolved the actin-binding cleft of DNASE1 (residues Tyr-65, Val-67 and Ala-114; G-actin Ki ≈ 5–10 nM) [8, 9], the C-terminal polybasic tail that renders DNASE1L3 both chromatin-competent and actin-resistant [10], and the histidine-based catalytic geometry that confines DNASE2 activity to acidic lysosomes [11, 12], providing a mechanistic scaffold for engineering therapeutic variants. Mechanistically, TREX1 has emerged as a tumor-cell-autonomous cytosolic checkpoint whose pharmacologic disruption sensitizes preclinical models to immune-checkpoint blockade [13–15], ADAR1 has been established as an RNA-editing gatekeeper of MDA5 [16], and the DC-expressed pool of DNASE1L3 has been shown to be a decisive determinant of neutrophil-extracellular-trap clearance in the tumor stroma [17]. Translationally, cell-free DNA fragmentomics has now been prospectively validated across 3,021 patients and 3,370 controls in 13 cancer types with area under the curve exceeding 0.98 [18]; the first selective ADAR1 small-molecule inhibitor (ADAR1i-124) [19] and dual-acting DNASE1/DNASE1L3 Fc-fusion biologics have entered late preclinical development; the RNase Fc-fusion RSLV-132 has completed a phase 2a randomised trial in lupus with signals of clinical benefit [20]; and JAK1/2 inhibition has become the effective standard of care in Aicardi–Goutières syndrome, supported by a 2026 multicentre controlled cohort [21, 22]. Collectively, these milestones underscore the need to systematically integrate these insights, thereby catalyzing the development of next-generation precision therapies.

A comprehensive review of contemporary high-impact studies reveals a critical convergence in the field of nucleic acid biology where the molecular identities of cytosolic sensors are well established yet current literature often treats nucleic acid metabolism as a housekeeping function distinct from immune surveillance. Building upon these insights, we posit a unifying framework that integrates these previously segregated concepts by proposing that self DNA and RNA exist in a dynamic equilibrium governed by two mutually exclusive fates, functioning either as potent danger signals decoded by specific sensor networks to trigger inflammation or as immunologically silent substrates that are rapidly sequestered and degraded before detection. Central to this dichotomy is the re-evaluation of nucleases which, rather than serving as passive garbage disposals, function as context-dependent editors that actively shape the immunological landscape and dictate a directional therapeutic duality essential for clinical intervention. This perspective suggests that in the context of oncology the objective is to restore ligand availability to expose malignancy to the immune system, whereas in autoimmunity the goal is to enhance clearance mechanisms to quell aberrant inflammation and restore tolerance. We substantiate this framework by dissecting the distinct biochemical grammars that separate the DNase and RNase families and by mapping how specific enzymatic perturbations redistribute nucleic acids between these fates to rationalize observed disease phenotypes and derive precise clinical strategies ranging from fragmentomics for diagnostics to enzyme replacement therapies. Ultimately, this synthesis aims to distinguish established consensus from working hypotheses, highlighting convergences and open priorities for future development in the therapeutic modulation of nucleic acid immunity. To develop this framework systematically, the review is organized into three linked parts that follow the logic of the title. Section 2 establishes the structural and biochemical grammar of the DNase and RNase families, substrate specificity, subcellular compartment and pH optimum, that determines which enzyme acts on a given nucleic acid and where. Section 3 maps this biochemistry onto the molecular mechanisms of immune sensing, linking the cGAS–STING, RIG-I/MDA5, OAS–RNase L and endosomal TLR axes to the nucleases that spatially restrain them and to the physical neutrophil-extracellular-trap layer. Sections 4 and 5 then translate this mechanistic picture into diagnostic and therapeutic strategy, first in cancer (Sect. 4) and then in non-malignant disease (Sect. 5), before the Discussion and Conclusion synthesize the two-fate framework and its open questions.

## Structural insights into nucleic-acid–clearing enzymes

**Fig. 1:**
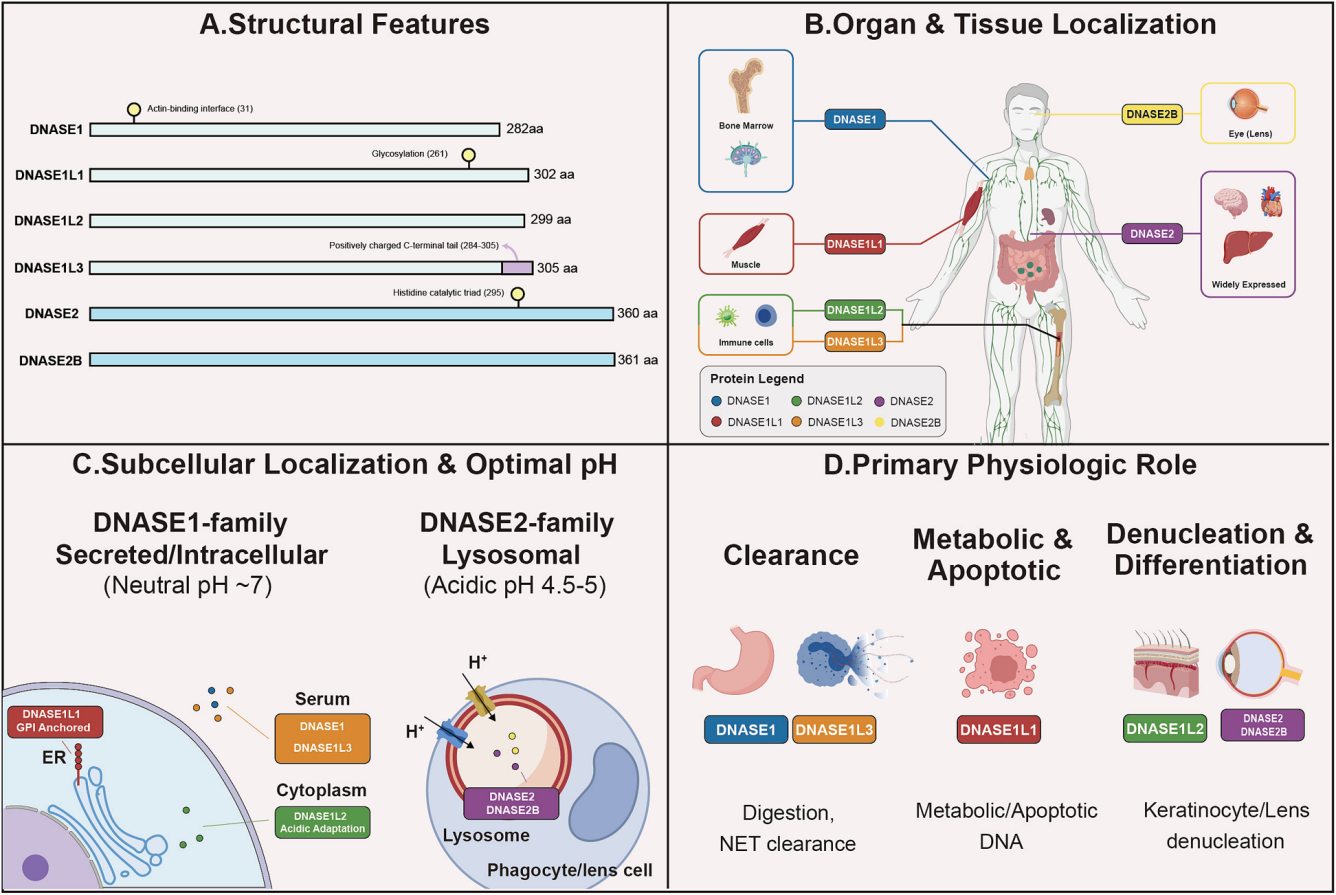
Structural, biochemical, and spatial heterogeneity of the mammalian DNASE family. **A** Schematic representation of the domain architectures and key biochemical features distinguishing the DNASE I-like and DNASE II superfamilies. Crucial structural motifs, including the actin-binding cleft of DNASE1, the membrane-anchoring glycosylation site of DNASE1L1, the polybasic chromatin-binding C-terminal tail of DNASE1L3, and the histidine-rich active site of DNASE2, are highlighted. **B** Anatomical and tissue-specific expression profiles of DNASE isoforms under homeostatic conditions, illustrating their functional compartmentalization. **C** Subcellular segregation defined by pH optima and metal-ion dependency. Cation-dependent DNASE I-like members (DNASE1, DNASE1L3) predominantly execute functions in neutral extracellular/cytosolic environments, whereas cation-independent DNASE II members operate within the acidic lumen (pH 4.5–5.0) of lysosomes. **D** Distinct physiological roles of specific DNASEs, ranging from dietary DNA digestion and neutrophil extracellular trap (NET) clearance to metabolic DNA scavenging and terminal differentiation (e.g., denucleation in keratinocytes and lens fiber cells)

Whether mislocalized self DNA and RNA activate or bypass the innate sensors is first decided by biochemistry: the same nucleic-acid substrate is either cleared before it reaches a sensor, cleaved into a sensor-engaging fragment, or preserved intact as a persistent danger signal, and the outcome depends on which nuclease acts in the surrounding compartment and at what pH. Substrate specificity, subcellular reach and pH optimum together determine which nucleases operate as extracellular guardians of DNA and RNA, which restrict intracellular nucleic-acid pools, and which function selectively in acidic lysosomes and it is this compartment-by-compartment division of labor that predicts whether a given self nucleic acid engages a downstream sensor or is silenced. The mammalian DNase family is organized into two superfamilies, namely the cation-dependent DNASE I–like group with a neutral pH optimum, and the cation-independent DNASE II family that operates in acidic compartments. The principal cytosolic and endosomal RNases form mechanistically distinct lineages with non-overlapping substrate preferences and pH optima. Each principal enzyme is examined below in the order in which its biochemistry constrains its function, moving from the extracellular and cytosolic DNases through the acidic-lysosomal hydrolases and then to the ribonuclease families that operate in parallel (Fig. 1, Table 1). Because both malignant and inflammatory disease arise from the same sensing–clearance imbalance acting in opposite directions, Table 1 summarizes each principal DNase and RNase along a common set of axes, gene locus, biochemical properties, substrate, tumor expression, the immune sensor engaged, and the key disease evidence, so that the cancer and autoinflammatory phenotypes can be read side by side.

**Table 1:** Comparative overview of the principal human DNase and RNase families in cancer and inflammatory disease biology

Member	Gene locus	Biochemical properties	Primary substrate	Expression in tumors	Immune sensor engaged	Key disease-related evidence	Representative animal models/clinical studies reference
DNASE1	16p13.3	Mg^2^⁺/Ca^2^⁺ dependent; neutral pH optimum	Extracellular dsDNA; NETs	↓ COAD, LUSC; ↑ KIRC, LIHC (paradoxical)	cGAS–STING (indirect, via NET-derived DNA persistence)	Actin-inhibited in necrotic cores; KIRC ↑ → poor OS	[10–12]
DNASE1L1 (DNASE X)	Xq28	Mg^2^⁺/Ca^2^⁺ dependent; GPI-anchored	Nuclear chromatin (apoptotic execution)	Overexpressed in multiple carcinomas (oral, breast, prostate, lung, colon, bladder) at protein level; nuclease activity blocked by tumor-specific inhibitors	None directly; Apo10 epitope serves as diagnostic readout rather than immune activator	Apo10 epitope = frustrated apoptosis; EDIM detects Apo10⁺ macrophages, sens > 90%/spec > 95%	[16, 17]
DNASE1L2	16p13.3	Mg^2^⁺/Ca^2^⁺ dependent; acidic pH optimum	Nuclear DNA during keratinocyte terminal differentiation	↑ Breast carcinoma (ectopic expression); variable in LUAD	Not established in cancer context	Ectopic ↑ in breast cancer → EMT; acidic pH advantage in TME	[18–20]
DNASE1L3 (DNASE γ)	3p14.3	Mg^2^⁺/Ca^2^⁺ dependent; actin-resistant; C-terminal basic tail	Chromatin in apoptotic microparticles; NET-associated DNA; nucleosomal DNA	↓ LIHC, LUAD, STAD, KIRC, BRCA (widespread but not universal downregulation; promoter hypermethylation or ZNF384-mediated silencing)	cGAS–STING (acute high-amplitude → IFN-β; chronic low-amplitude → NF-κB/IL-6)	↓ → microparticle DNA + chronic STING/NF-κB; DC-DNASE1L3 NET clearance; restoration → PANoptosis + αPD-1/sorafenib synergy; ↓ OS across KIRC/LIHC/LUAD/PAAD	[22–27]
DNASE2	19p13.2	Cation-independent; acidic pH optimum (pH 4.5–5.0)	Lysosomal DNA from phagocytosed apoptotic cells and autophagic cargo; RNA (dual substrate)	↑ GBM, LGG, PAAD, melanoma; correlates positively with tumor grade and macrophage infiltration, inversely with T cell markers	cGAS–STING (suppressed by DNASE2-mediated lysosomal containment of DNA)	GBM ↑ → T cell exclusion, poor OS; lysosomal DNA escape block; inhibitor → cold-to-hot conversion; Mn007 = promiscuous aggregator	[28–30]
DNASE2B	1p31.1	Cation-independent; acidic pH optimum	Nuclear DNA during lens fiber cell differentiation	Ectopic expression in LUAD (context-dependent prognostic value); restricted expression in other tumor types	Not established	Ectopic ↑ in LUAD (favorable in some cohorts); efferocytosis aid; differentiation co-option	[29]
RNase L	RNASEL 1q25.3	Mg^2^⁺ dependent; ATP-independent; activation gated by 2′,5′-oligoadenylate (2-5A) binding and dimerization	Single-stranded RNA at UA/UU dinucleotides; cleavage yields 5′-OH and 2′,3′-cyclic phosphate termini that themselves act as RIG-I ligands	Constitutively expressed; activity gated by OAS sensing of dsRNA; OAS1/2/3 expression altered in breast cancer cohorts	RIG-I (re-engaged via cleavage products in an autocatalytic loop); 2-5A also transfers between cells to extend the antiviral state community-wide	Cross-cell 2-5A transfer → community antiviral state; SKIV2L brake; OAS/RNase L variants ↔ COVID-19 severity	[31–33]
RNase H1	RNASEH1 2p25.3	Mg^2^⁺/Mn^2^⁺ dependent; neutral pH; nuclear and mitochondrial isoforms	RNA strand of RNA:DNA hybrids; endonucleolytic; requires ≥ 4 consecutive ribonucleotides	Ubiquitous; not systematically dysregulated as a cancer driver in current evidence	Indirect via R-loop biology: prevents accumulation of RNA:DNA hybrids that would otherwise yield cytosolic ligands for cGAS–STING/MDA5	Essential for mtDNA replication; R-loop biology in oncology contextual	[34]
RNase H2	RNASEH2A 19p13.13; RNASEH2B 13q14.3; RNASEH2C 11q13.1 (heterotrimeric)	Mg^2^⁺/Mn^2^⁺ dependent; neutral pH; trimer required for activity	RNA:DNA hybrids and ribonucleotides misincorporated into genomic DNA (ribonucleotide excision repair); resolves R-loops at replication–transcription conflicts	Upregulated under replication stress in multiple cancers; targeted dependency described in TNBC and BRCA1/2-mutant tumors	cGAS–STING (cytosolic RNA:DNA hybrid and DNA leakage on loss); MDA5 indirectly via cytosolic RNA species	Tumor ↑ buffers replication stress; TNBC ↓ + PARPi → STING; biallelic LoF → AGS	[34–36]
RNase T2	RNASET2 6q27	Cation-independent; acidic pH optimum (pH 4.5–5.5); endolysosomal localization	Single-stranded and structured RNA in the endolysosome; transferase + hydrolase activity; preferentially cleaves at purines; major endolysosomal RNase functionally analogous to DNASE2 on the DNA side	Variable across tumor types; no consistent tumor driver role in current evidence	Endolysosomal RNA processing supporting TLR8 substrate generation; failure releases lysosomal RNA into the cytosol	Biallelic LoF → leukoencephalopathy (cCMV-like); zebrafish rRNA accumulation; mouse type I IFN brain	[36]

### DNASE1: canonical extracellular guardian and its actin liability

DNASE1 (16p13.3) is a secreted glycoprotein that serves as the predominant nuclease in extracellular fluids [23]. Catalysis requires Mg^2^⁺ and Ca^2^⁺, ensuring high serum efficiency while restricting cytoplasmic activity [8]. A defining feature for cancer biology is the high-affinity inhibition of DNASE1 by monomeric actin, or G-actin, released during tumor necrosis. G-actin binds with Ki ≈ 5–10 nM and occludes the catalytic cleft [37], with the interaction involving residues Tyr-65, Val-67, and Ala-114 [9]. Because extracellular G-actin in necrotic regions exceeds 100 nM, this complex forms readily in vivo, creating a nuclease-deficient zone in which immunogenic DNA debris and NETs persist [24]. This actin liability has motivated the development of actin-resistant DNASE1 variants for therapeutic use [38]. Actin sequestration is the Achilles heel of DNASE1; DNASE1L1 addresses part of this liability by relocating to the ER and nuclear envelope, at the cost of surrendering direct extracellular access.

### DNASE1L1 (DNASE X): the GPI-anchored sentinel and the Apo10 paradox

DNASE1L1 (Xq28) is homologous to DNASE1 but tethered to the ER and nuclear envelope through an N-glycosylation site [28, 39]. In cancer, dysregulated apoptosis, in particular IAP-driven blockade of execution steps, causes nuclear DNASE1L1 to accumulate without effective DNA degradation, generating the Apo10 epitope [29, 40]. This "frustrated apoptosis" signature underpins the EDIM diagnostic platform, in which macrophages serve as biosensors for tumor-derived Apo10. Whereas DNASE1L1 solves the compartmentalization problem by tethering to intracellular membranes, DNASE1L2 illustrates the alternative strategy of restricting expression to a single tissue and repurposing pH tolerance for specialized differentiation programs.

### DNASE1L2: the keratinocyte-specific remodeler

DNASE1L2 (16p13.3) arose by gene duplication from DNASE1 and acquired a restricted expression profile in epidermal keratinocytes, where it mediates nuclear DNA degradation during terminal cornification, and loss causes parakeratosis [31, 32]. In breast carcinoma, ectopic DNASE1L2 upregulation correlates with stem-like phenotypes, E-cadherin suppression, and EMT activation, suggesting that tumor cells repurpose its chromatin-modifying capacity for plasticity [33]. The acidic pH optimum of DNASE1L2 supports a biochemically plausible working hypothesis of activity in the acidified tumor microenvironment, but direct in situ enzyme activity measurements are still lacking [41]. DNASE1L2 shows how tissue-restricted specialization can constrain a DNase to a single differentiation program; DNASE1L3, in contrast, is broadly expressed by professional immune cells and acquires its distinctive function through a structural addition to the DNase I fold.

### DNASE1L3 (DNASE Gamma): the C-Terminal tail determines function

DNASE1L3 (3p14.3) is distinguished from DNASE1 by a positively charged C-terminal peptide tail that binds lipid membranes and nucleosomal DNA [10], and confers two unique capacities, namely degradation of chromatin shielded by histones and lipid bilayers [34], and intrinsic resistance to actin inhibition due to absent salt-bridge interactions [35]. We use "membrane penetration" operationally to denote access to membrane-protected substrates, since direct biochemical demonstration through vesicle-leakage assays remains a methodological gap [10]. Secreted predominantly by dendritic cells and macrophages, DNASE1L3 functions as an extrinsic tumor suppressor [36], and its loss, whether through promoter hypermethylation or deletion, is a recurrent feature of solid malignancies and a driver of lupus-like autoimmunity [42]. Engineering efforts now seek to combine DNASE1 stability with DNASE1L3 potency. The DNASE I–like enzymes collectively handle nucleic-acid substrates in the extracellular and cytosolic space; the DNASE II family occupies the complementary lysosomal niche, where acidic pH and cation-independent catalysis define an entirely different operating envelope.

### DNASE2 and DNASE2B: the lysosomal acidic nucleases

DNASE2 (19p13.2) and DNASE2B (1p31.1) are metal-independent nucleases optimized for the lysosomal lumen at pH 4.5–5.0 [11]. DNASE2 is abundant in phagocytes for apoptotic-material clearance [43], whereas DNASE2B is restricted to the ocular lens. Notably, DNASE2 also degrades RNA [44], so it bridges the DNA and RNA arms and is more accurately a broad-spectrum endolysosomal nuclease. In oncology, tumor cells exploit this capacity to degrade autophagic cargo and dampen cGAS–STING, and upregulation in glioblastoma reinforces the immunologically cold phenotype [12]. The medicinal-chemistry obstacles to DNASE2 inhibition and its immunopathological risks are addressed when we discuss DNASE2 as a druggable target in cancer. This completes the DNase family survey; the RNase families reviewed next mirror the same organizing logic of substrate access, subcellular compartment and pH optimum, with distinct lineages functionally aligned to DNASE1L3, DNASE2 and TREX1 on the DNA arm.

### The core ribonuclease families: RNase L, RNase H, RNase T2

Three RNase lineages dominate immune-relevant RNA turnover in mammalian cells and operate as functional counterparts to DNASE1L3, DNASE2, and TREX1, respectively. RNase L is the principal cytosolic ssRNA endonuclease engaged by the OAS–2-5A pathway. Pathogen-derived dsRNA activates OAS1, OAS2, and OAS3, which synthesize 2-5A, and 2-5A binding dimerizes RNase L and licenses cleavage at UA/UU dinucleotides in host and viral RNAs. The cleavage products themselves act as RIG-I ligands, generating an autocatalytic amplification loop, and 2-5A can transfer between cells in a paracrine manner to extend the antiviral state community-wide [45, 46]. The cytoplasmic helicase SKIV2L extracts cleavage products and limits chronic RNase L activation [47]. RNase H1 and RNase H2 resolve RNA:DNA hybrids and remove ribonucleotides misincorporated into genomic DNA. RNase H2 is heterotrimeric, encoded by RNASEH2A, RNASEH2B, and RNASEH2C, performs the bulk of nuclear ribonucleotide excision repair, and resolves R-loops at replication–transcription conflicts [30, 48]. Loss-of-function in any subunit causes Aicardi–Goutières syndrome through cytosolic RNA:DNA hybrid leakage and cGAS–STING engagement engagement, a mechanistic thread we return to when discussing AGS. In cancer, tumors upregulate RNase H2 to relieve replication stress, and depletion in TNBC sensitizes to PARP inhibition while activating cGAS–STING [49, 50]. RNase T2 is the acidic endolysosomal RNase functionally analogous to DNASE2 for DNA, and biallelic RNASET2 loss causes a leukoencephalopathy with lysosomal rRNA accumulation [51, 52]. The cytosolic 3' → 5' exoribonuclease REXO2 and DIS3L2 clear short RNA species that could otherwise activate cytosolic RNA sensors [53, 54]. The structural specializations catalogued above resolve into a single mechanistic chain that connects nuclease biochemistry to cGAS activation and to the diagnostic reach of plasma cfDNA. In DNASE1, the actin-binding cleft creates a low-nanomolar susceptibility to circulating G-actin (K_i ≈ 5–10 nM) that suppresses its chromatin-directed activity in cancers with high tumor-cell necrosis [8, 9]; the actin-resistant DNASE1L3, by contrast, retains chromatin-directed activity in the same tumor microenvironments and produces a characteristic short-fragment ladder in plasma cfDNA that is measurable through nucleosome-positioning and end-motif profiling [25]. This short-fragment ladder crosses the ~40 bp cGAS-activating threshold asymmetrically across DNase family members [55, 56], so the identity of the acting enzyme determines whether cfDNA output engages the sensor or is filtered below detection. The chemistry at fragment termini reinforces this hierarchy: DNASE1 and DNASE1L3 generate 5′-phosphate and 3′-hydroxyl ends whose accessibility supports downstream sensing, while DNASE2 hydrolysis at lysosomal pH yields 5′-hydroxyl and 3′-phosphate ends whose intracellular fate is degradative rather than sensor-engaging. The structure-to-fragmentomics-to-cGAS chain therefore closes the biochemistry into the mechanistic architecture developed in the next section.

## Molecular mechanisms linking nucleic-acid clearance to immune sensing

**Fig. 2:**
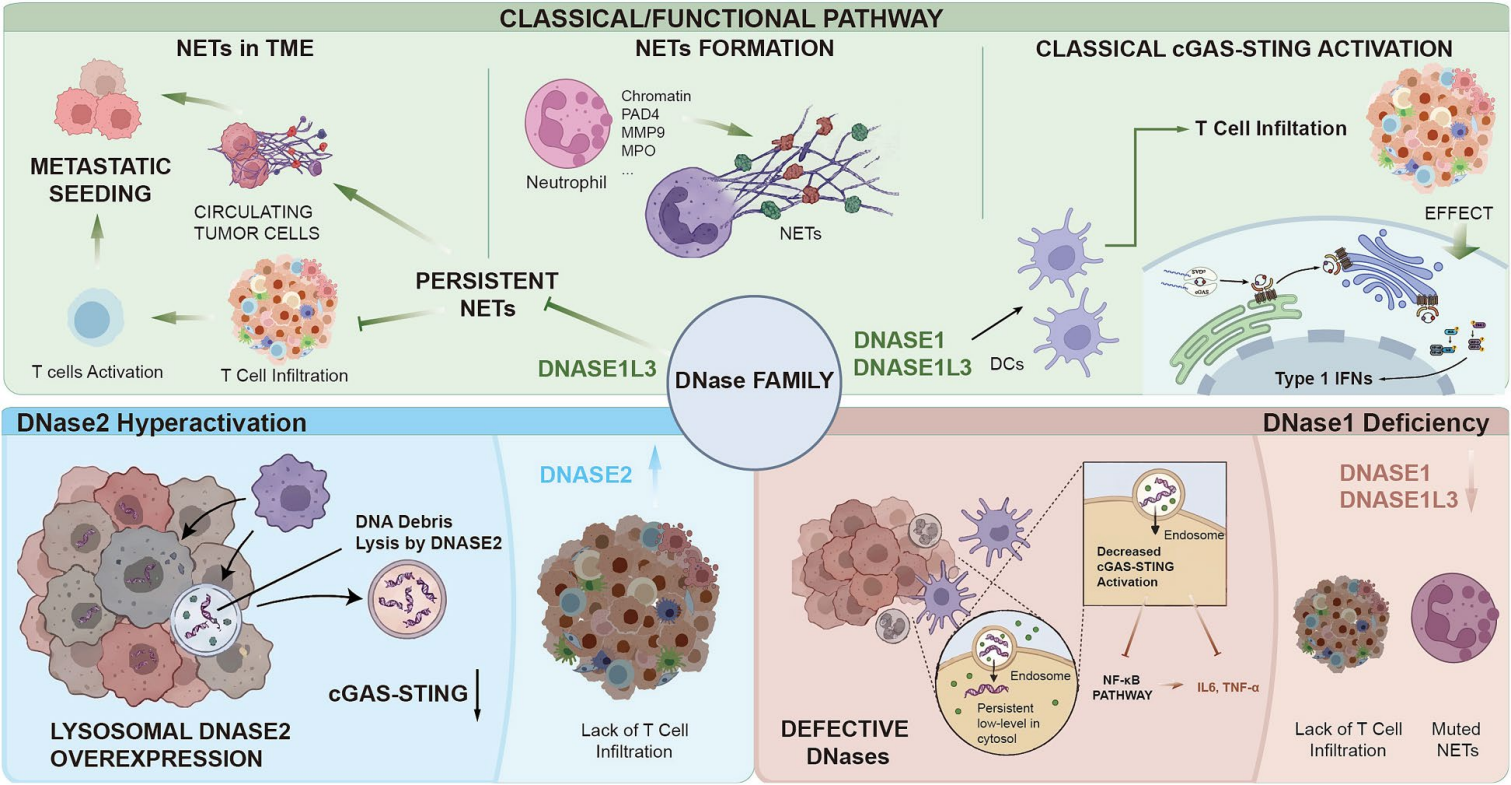
Spatiotemporal regulation of the cGAS-STING innate immune sensing axis by the DNases family in the tumor microenvironment (TME). **A** Canonical cGAS-STING activation. Under conditions of acute physiological stress or infection, excessive cytosolic double-stranded DNA (dsDNA) binds and activates cGAS, generating the second messenger cGAMP. cGAMP subsequently activates ER-resident STING, leading to the recruitment of TBK1 and the phosphorylation of IRF3. This acute, high-amplitude signaling cascade translocates to the nucleus to drive the robust expression of Type I interferons (IFNs), effectively orchestrating CD8⁺ T cell and natural killer (NK) cell activation. **B** DNASE1L3 deficiency-mediated chronic inflammation. In malignancies such as hepatocellular carcinoma (HCC), lung adenocarcinoma (LUAD), and colon adenocarcinoma (COAD), the downregulation of DNASE1L3 enables the accumulation of chromatin-laden apoptotic microparticles. Following endocytosis by tumor cells or dendritic cells (DCs), persistent, low-level DNA leakage from endosomes into the cytosol triggers chronic STING activation. This prolonged, sub-lethal STING signaling selectively diverts the downstream cascade toward a non-canonical NF-κB pathway, resulting in the predominant secretion of pro-tumorigenic cytokines (IL-6, TNF-α). This inflammatory milieu actively recruits myeloid-derived suppressor cells (MDSCs) and regulatory T cells (Tregs), fostering an immunosuppressive TME. **C** Immune silencing via DNASE2 overexpression. In highly aggressive, immunologically "cold" tumors like glioblastoma multiforme (GBM) and pancreatic adenocarcinoma (PAAD), tumor cells and tumor-associated macrophages (TAMs) frequently overexpress lysosomal DNASE2. This enzyme rapidly and exhaustively hydrolyzes massive amounts of DNA debris engulfed via phagocytosis or macroautophagy. By preventing any cytosolic leakage of un-degraded DNA fragments, DNASE2 effectively establishes an "immunological blind spot," preemptively extinguishing cGAS-STING activation and maintaining a stealth phenotype characterized by a profound lack of T cell infiltration

**Fig. 3:**
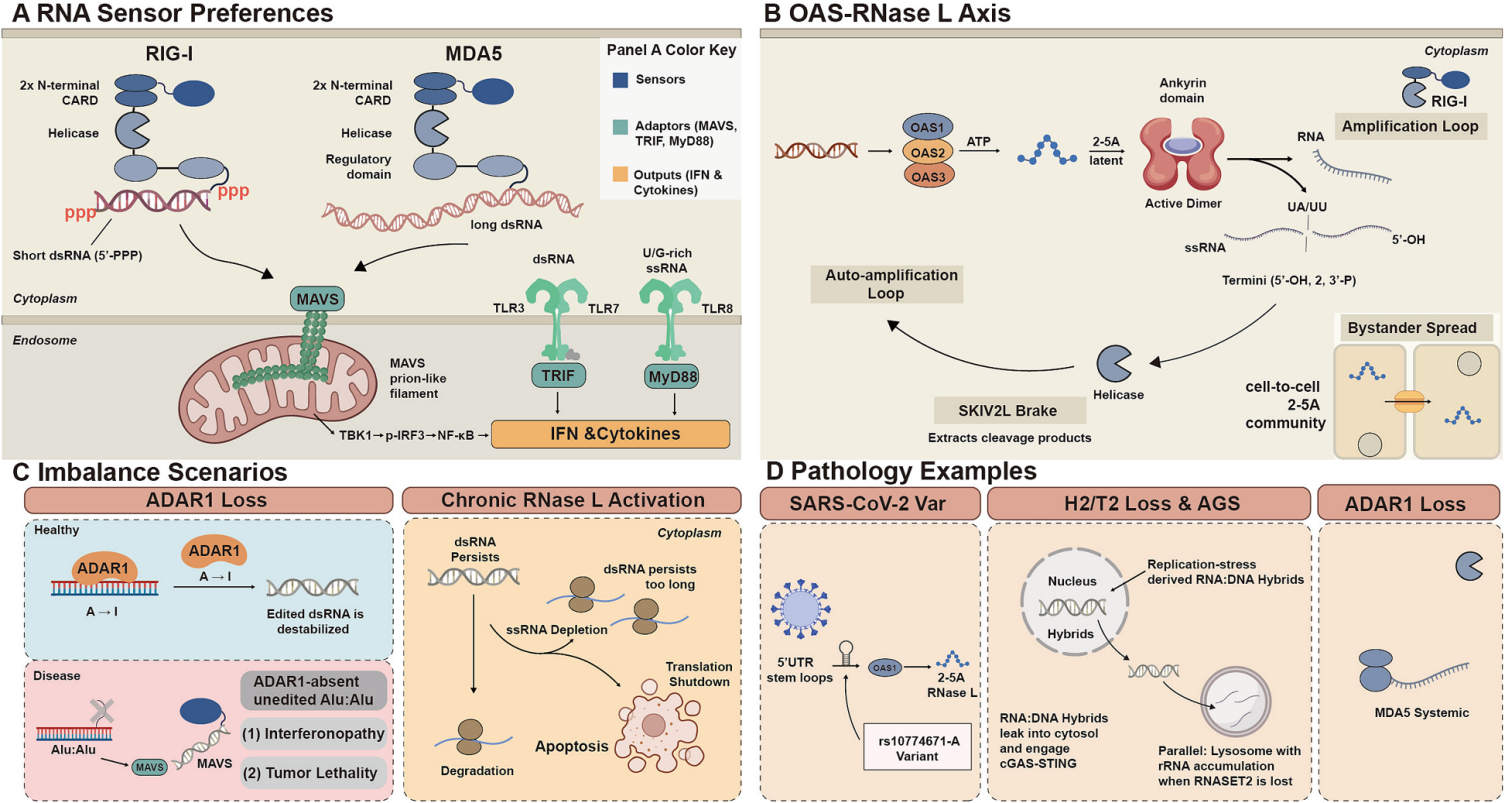
The RNA arm of nucleic acid sensing and clearance. **A** Canonical RNA sensing. Cytosolic double-stranded RNA (dsRNA) bearing 5'-triphosphate engages RIG-I (short dsRNA, 10–300 bp) or MDA5 (long dsRNA, > 1–2 kb), while endosomal dsRNA and uridine-rich single-stranded RNA engage TLR3 and TLR7/8 respectively. RIG-I and MDA5 signal through the mitochondrial adaptor MAVS, which polymerizes into prion-like filaments and activates TBK1, IRF3, and NF-κB to drive type I IFN and pro-inflammatory cytokine output. **B** The OAS–RNase L axis. Engagement of OAS1/2/3 by dsRNA generates the second messenger 2-5A, which dimerizes and activates the latent endoribonuclease RNase L. RNase L cleaves single-stranded host and viral RNA at UA/UU dinucleotides, producing 5'-OH/2',3'-cyclic phosphate ends; these short fragments re-engage RIG-I, creating an autocatalytic amplification loop. The helicase SKIV2L counterbalances RNase L by extracting cleavage products. Paracrine 2-5A trafficking extends antiviral signaling to neighboring cells. **C** Imbalance scenarios. Loss of ADAR1 unmasks endogenous Alu:Alu dsRNA and triggers MDA5-driven interferonopathy in humans and mice, or in BRCA1/2-mutant tumors, MDA5-mediated tumor cell death after pharmacologic ADAR1 disruption. Chronic OAS–RNase L activation depletes the cytosolic RNA pool and promotes apoptosis. **D** Non-tumor pathology examples. SARS-CoV-2 5'-UTR stem-loops are strong OAS1 activators; common variants reducing OAS1 protein abundance (rs10774671-A) are associated with COVID-19 hospitalization. RNASEH2A/B/C and RNASET2 loss-of-function mutations cause Aicardi–Goutières syndrome through accumulation of unresolved RNA:DNA hybrids and lysosomal rRNA, respectively, converting an RNA-axis defect into cGAS–STING-driven type I IFN signaling. ADAR1 loss-of-function produces a lethal MDA5-driven type-I interferonopathy. Together, panels A–D mirror the cGAS–STING–DNase logic of Fig. 2 on the RNA arm and underscore that disease-relevant imbalance can occur at the sensor level (RIG-I/MDA5/TLR), the clearance level (RNase L/RNase H2/RNase T2), or the editing level (ADAR1)

The structural grammar developed in the preceding chapter now becomes actionable. Each biochemical specialization catalogued above finds its meaning at the interface where self DNA and self RNA meet the innate sensors, and that interface is what the manuscript title designates the molecular mechanisms of DNA and RNA immunity: the set of enzymatic and sensor-side processes by which the same endogenous nucleic acid either accumulates to a sensor-engaging threshold and drives an inflammatory program, or is cleared, edited or sequestered below that threshold and remains immunologically silent. Three tiers of mechanism together determine which fate obtains, and this chapter is organised around them. First and central is the cGAS–STING axis on the DNA side and the RIG-I/MDA5, OAS–RNase L and endosomal TLR3/7/8 receptors on the RNA side, whose ligand thresholds and signal duration establish where the fate switch sits; alongside these principal sensors, AIM2 and TLR9 offer parallel DNA-side readouts whose relative contributions in cancer and autoimmunity we frame as a working hypothesis where direct human evidence is limited. Second are the nucleases that spatially restrain these sensors, the TREX1, DNASE1L3 and DNASE2 trinity on the DNA arm, together with ADAR1 on the RNA arm, whose loss-of-function, gain-of-function or actin-mediated inactivation is the principal disease-driving perturbation and therefore the primary therapeutic target of the chapters that follow. Third is the physical NET layer, where the DNASE1/DNASE1L3 balance and complement C1q–DNASE1 crosstalk determines whether extracellular chromatin persists as a barrier to immune infiltration and as a chronic sensor ligand. The DNA-arm framework is illustrated in Fig. 2 and the RNA-arm framework in Fig. 3, and throughout we distinguish established consensus from working hypotheses supported by indirect or model-system evidence.

### cGAS–STING as the Core DNA-sensing axis

cGAS is the master cytosolic DNA sensor that couples the presence of mislocalized DNA to type I interferon output. Ligand binding is length-dependent, requiring approximately 40 base pairs of double-stranded DNA to organize the ladder-like cGAS oligomer that generates the second messenger 2′3′-cyclic GMP-AMP (cGAMP) [55, 56]. The length threshold is biologically important because it discriminates chromatin-scale danger from small physiological DNA remnants, and it explains why nuclease activity that fragments cytosolic DNA below the threshold effectively silences the pathway. Two parallel DNA sensors, AIM2, which engages an inflammasome program [57], and TLR9, which detects unmethylated CpG in the endosome operate alongside cGAS with distinct downstream outputs, and the relative contribution of each sensor to specific tumor and autoimmune contexts remains an open question that we address in the Discussion. cGAMP is a diffusible second messenger that binds STING at the endoplasmic reticulum, triggering a stereotyped trafficking sequence in which activated STING moves from the ER through the ERGIC to the Golgi and undergoes autophagy-linked degradation on a defined timescale [58]. During transit, STING recruits and activates TBK1, which phosphorylates IRF3 to drive type I IFN transcription and, in parallel, activates the IKK complex to induce NF-κB–driven pro-inflammatory cytokines [59]. Signal duration and amplitude appear to bias output between these two arms: acute high-amplitude signaling favors TBK1–IRF3–IFN, whereas chronic low-amplitude signaling favors IKK–NF-κB and cytokine skewing toward IL-6 and TNF-α, a distinction that becomes central in the tumor and autoimmune contexts addressed below. The amplitude-dependent pathway-selection model itself remains a working hypothesis (Grade C) supported by indirect evidence and is developed further in the DNASE1L3-deficiency discussion [60, 61]. The downstream immune output of cGAS–STING activation is a coordinated program that primes CD8⁺ T cells, recruits NK cells, licenses dendritic cell cross-presentation and, at sustained low levels, remodels the myeloid compartment toward suppression [62]. This dichotomy sets up the central problem developed in the remainder of this mechanistic chapter: nucleases are the principal molecular brake on cGAS–STING activation, and their loss, gain or actin-mediated inactivation determines whether the pathway operates as a physiologic guardian or a pathogenic accelerant. Having established the sensor and the compartmental logic that makes localisation determinative, the immediate next step is to name the three enzymes that spatially restrain cGAS in vivo, since the loss-of-function and gain-of-function scenarios developed further in this chapter act predominantly on this trinity.

### The spatial trinity of cGAS-restraining nucleases: TREX1, DNASE1L3 and DNASE2

Immediately downstream of cGAS lies a spatially distributed trinity of nucleases, TREX1, DNASE1L3 and DNASE2, that together delineate the full geometry of cGAS restraint and set the compartmental thresholds at which self DNA either engages the sensor or is cleared before it does. Compartmentally, TREX1 patrols the cytosol as the dominant 3′ → 5′ exonuclease acting on ssDNA and dsDNA generated by replication stress and reverse transcription of retroelements [63], DNASE1L3 acts extracellularly and on endolysosomal chromatin [34], and DNASE2 hydrolyzes DNA within acidic lysosomes after phagocytosis or macroautophagy [64]. In substrate specificity, TREX1 is a non-processive 3′-overhang exonuclease optimized for ssDNA [65], DNASE1L3 is a duplex endonuclease with acquired chromatin and lipid-bilayer competence [66], and DNASE2 is a broad-specificity endonuclease with a pH optimum of 4.5–5.0. In catalytic architecture, TREX1 uses a single-Mg^2^⁺ active site [67], DNASE1L3 uses the canonical two-metal-ion DNASE I–like fold, and DNASE2 is metal-independent with a histidine-based mechanism. Each enzyme therefore intercepts cytosolic DNA at a different point in the trafficking pathway that would otherwise deliver ligand to cGAS. The disease footprints of these three enzymes reflect this spatial division. Biallelic TREX1 loss-of-function produces Aicardi–Goutières syndrome type 1, in which cytosolic ssDNA accumulates and drives sustained cGAS–STING–IFN signaling, and TREX1 patients phenocopy Trex1-null mice, providing rigorous human evidence that loss of a cytosolic nuclease is causally sufficient for interferonopathy [63, 68, 69]. Biallelic DNASE1L3 loss produces familial hypocomplementemic urticarial vasculitis–like SLE with anti-chromatin autoantibodies and severe glomerulonephritis [70, 71], and reduced serum DNASE1L3 activity or anti-DNASE1L3 autoantibodies also mark a subset of sporadic SLE [34], patterns we return to when discussing SLE. DNASE2 loss in mice is embryonically lethal from unresolved macrophage-engulfed DNA driving fetal interferonopathy [43, 72], and heterozygous or hypomorphic DNASE2 variants in humans have been associated with an AGS-like presentation with anti-dsDNA antibodies; the human disease evidence remains sparse compared with the mouse literature, and we flag this asymmetry as an important interpretive caveat. The therapeutic direction that follows from this comparative anatomy is the central logic of the review. TREX1 has emerged as a tumor-cell-autonomous cytosolic immune checkpoint whose pharmacologic disruption induces cGAS-driven type I IFN, sensitizes preclinical tumors to checkpoint blockade, and yields durable regression across pancreatic, breast and melanoma models [15, 73], with selective small-molecule TREX1 inhibitors advancing through late preclinical development and forming a principal therapeutic vector in oncology as we develop further in the therapeutics section. DNASE1L3 follows the opposite direction in autoimmunity: engineered actin-resistant DNASE1/DNASE1L3 Fc-fusion biologics are designed to replace lost extracellular activity, restore microparticle chromatin clearance and dampen chronic sensor engagement [9, 74]. DNASE2 occupies the most ambivalent position: in tumors, DNASE2 inhibition is a conceptually attractive way to force lysosomal DNA overflow into the cytosol and reawaken cGAS–STING, but current tool compounds such as Mn007 act via non-specific colloidal aggregation rather than active-site binding [75], and selective inhibitors remain a medicinal-chemistry frontier rather than a translational reality. Read together, the three enzymes exemplify the directional duality that motivates this review, and the malignant reconfiguration of each of them, loss of DNASE1L3, gain of DNASE2 and reactivation of TREX1, is the specific perturbation that carries the framework into the tumor microenvironment; the loss-of-function pole is examined immediately below, in the tumor biology of DNASE1L3 downregulation.

### DNASE1L3 deficiency: the trap of chronic inflammation

Across many malignancies, DNASE1L3 downregulation shifts cytosolic DNA sensing from acute IFN to chronic maladaptive signaling. Loss of DNASE1L3 permits accumulation of DNA-laden microparticles protected from extracellular degradation [34], and Following endocytosis, DNA escapes into the cytoplasm via mechanisms that remain mechanistically underspecified in the absence of DNASE1L3. By analogy with related lysosomal DNA-stress and phagocytic dsDNA-delivery systems, three non-mutually-exclusive candidates have been proposed: endosomal membrane rupture, channel-protein–mediated translocation, and impaired phagosome/lysosome maturation. While the former has been documented for phagocytic dsDNA delivery through processes such as NADPH-oxidase–dependent lipid peroxidation and DNGR-1-triggered rupture within the 1–1.5 μm size range [26], the latter is established as a permissive condition for DNA leakage in models of lysosomal-catabolism defects and lupus susceptibility [27, 76]. However, none of these mechanisms has been directly resolved in the specific context of DNASE1L3 loss-of-function. While physiological clearance triggers a rapid type I IFN response that supports antitumor immunity [58], DNASE1L3 deficiency causes persistent low-level DNA exposure and chronic STING activation. Signaling amplitude appears to dictate pathway selection. Acute signaling favors TBK1–IRF3, whereas chronic signaling promotes IKK–NF-κB, shifting cytokine output to pro-tumorigenic IL-6 and TNF-α and recruiting MDSCs and Tregs [77, 78]. DNASE1L3-knockout mice develop larger colorectal tumors with reduced CD8⁺ T cell infiltration. The "chronic low-amplitude STING preferentially engages NF-κB" model (Grade C) remains a working hypothesis supported by indirect evidence, and we return to its evidentiary status below. Whether the DNASE1L3 shortfall in these tumors arises from tumor-cell-autonomous silencing or from a loss of intratumoral dendritic-cell-derived enzyme is a cellular-origin question to which we return in the Discussion. DNASE1L3 downregulation illustrates the loss-of-function pole of nuclease dysregulation, a passive loss of restraint that permits ligand accumulation and chronic sensor drive. Its mirror image is an active silencing strategy, in which tumor cells co-opt a different member of the trinity to reinforce lysosomal degradation and thereby cut off cGAS ligand supply, and this gain-of-function configuration is realised most conspicuously through DNASE2 over-expression.

### DNASE2 overexpression: the mechanism of immune silencing

Prominent examples of human tumoral tissues in which this active DNASE2-driven silencing has been documented include glioblastoma and pancreatic adenocarcinoma, both of which have been associated with elevated DNASE2 expression that reinforces immune silence (Grade C). Facing nucleic acid stress from rapid turnover and genomic instability, these tumors are proposed to use DNASE2 as a lysosomal gatekeeper, where rapid hydrolysis of DNA after autophagy or phagocytosis prevents cytosolic escape and constrains cGAS–STING [79]. In glioblastoma, high DNASE2 correlates with reduced T cell infiltration and poor survival [43]. DNASE2-deficient mice suffer severe anemia and inflammation driven by uncontrolled type I IFN. Pharmacologic DNASE2 inhibition in TAMs is a therapeutic objective, since it should restore cytosolic DNA accumulation and STING signaling [75], but the medicinal-chemistry obstacles and immunopathological risks of systemic DNASE2 blockade are non-trivial when we address DNASE2 as a druggable target in cancer. Two evidentiary limits temper this reading and must be stated before the therapeutic argument advances. The available human evidence is drawn from immunohistochemical and gene-expression studies of tumor tissue, which cannot establish the tumor-cell-autonomous requirement for DNASE2 in this silencing program in the absence of matched patient-derived knockdown data. Building on that same evidentiary asymmetry, the causal claim that tumor-cell DNASE2 over-expression directly suppresses STING signaling remains a working hypothesis supported by correlative evidence rather than an established mechanism, and this distinction is preserved throughout the Discussion. Loss and gain of DNase activity operate at the molecular level; the same biology is compounded by a physical layer of immune evasion when neutrophil extracellular traps escape clearance.

### The NET physical layer: DNASE1/DNASE1L3 balance and complement C1q crosstalk

The extracellular physical layer of self DNA in disease is the neutrophil extracellular trap, and the DNase system regulates it directly [80]. Neutrophil PAD4 catalyses histone citrullination and chromatin decondensation [81], and the released web is decorated with elastase, MMP9 and MPO [82]. In distant microvasculature such as the lung and liver, NETs sieve circulating tumour cells and increase metastatic colonisation efficiency [82], embedded proteases degrade the ECM to facilitate extravasation, and exposed DNA filaments engage receptors such as CCDC25 to activate proliferative signalling [83]. NETs additionally contribute to dormant tumour reactivation and recurrence [84–87], and a comprehensive synthesis of the NET literature in cancer, infection and autoimmunity has recently appeared [88–93]. Clearance of this layer depends on a two-enzyme balance. In healthy tissue, circulating DNASE1 dissolves intravascular NETs while DNASE1L3 handles interstitial and systemic chromatin; in cancer DNASE1 is frequently inhibited by elevated G-actin from necrotic tumour tissue, permitting prolonged NET stability [66, 94]. DNASE1L3-expressing dendritic cells dismantle NETs around tumour nests [17], and DNASE1L3 absence leads to dense NET networks that impede CD8⁺ T-cell access, a mechanism underlying immune-checkpoint resistance [95–97]. The operative mechanisms on this substrate are twofold and mutually reinforcing: physical shielding of tumour cells from cytotoxic effectors through blockade of T-cell migration and direct contact, together with local sequestration of chemokines and granule effector molecules within citrullinated chromatin, and both contribute to immunotherapy resistance in DNASE1L3-low tumours. The complement protein C1q binds chromatin and citrullinated histones with high affinity and partially protects decorated NETs from DNASE1 digestion [98, 99], so in SLE and antiphospholipid syndrome this protection contributes to NET persistence even with normal DNASE1 activity because C1q-coated chromatin is degraded slowly and continues to engage cGAS [100]; C3a and C5a additionally promote NETosis, creating a feed-forward circuit, and in tissues with prominent complement deposition, inflamed glomeruli, atherosclerotic lesions and the metastatic niche, DNASE1 must compete with C1q for NET access. Combined DNase replacement with anti-C5 or C1q-blocking agents is now an active preclinical strategy [101].

### The RNA Sensing arsenal: RIG-I, MDA5 and Endosomal TLR3/7/8

The DNA-sensing logic operates in parallel with a structurally distinct RNA arsenal. RIG-I, encoded by DDX58, is a cytosolic helicase preferring short dsRNA of 10–300 bp bearing 5'-triphosphate, and ligand engagement triggers CARD-mediated oligomerization, MAVS polymerization on mitochondria, and TBK1/IRF3/NF-κB activation [102]. MDA5, encoded by IFIH1, recognizes long dsRNA filaments exceeding 1–2 kb and signals through the same MAVS hub, but with a higher activation threshold, so endogenous dsRNA must be edited or destabilized to escape detection [16], as we develop in the OAS–RNase L subsection that follows. The endosomal sensors TLR3, TLR7, and TLR8 recognize dsRNA and U/G-rich ssRNA, signaling through TRIF or MyD88. Selective TLR7/8/9 modulators with applications in oncology and chronic inflammation are now in development [103–105]. The protective-vs-pathologic duality of RNA sensors closely parallels the cGAS–STING dichotomy. Having named the RNA-side sensors, the ligand-clearance question that dominates the DNA arm returns in its RNA-arm form: which enzymatic system regulates cytosolic RNA availability so that these receptors are engaged when it matters and silenced when it does not. That system is the OAS–RNase L cascade, whose position on the RNA arm mirrors the position DNASE1L3 occupies on the DNA arm.

### The OAS–RNase L Axis: an RNA-side counterpart to DNASE1L3

Cytosolic dsRNA engages OAS1, OAS2, and OAS3, which synthesize 2-5A. 2-5A then dimerizes and activates RNase L, which cleaves ssRNA at UA/UU dinucleotides, depleting the cytosolic RNA pool, halting protein synthesis, and, when sustained, initiating apoptosis [46]. The cleavage products bear 5'-OH and 2',3'-cyclic phosphate termini and re-engage RIG-I, creating an autocatalytic loop linking RNA clearance back to RNA sensing [45]. 2-5A trafficking between cells generates community-wide antiviral states and explains bystander effects in viral infection. The cytoplasmic helicase SKIV2L extracts RNase L products and brakes chronic activation [47], and its loss produces a trichohepatoenteric syndrome with interferonopathy features. In oncology, OAS variants and RNase L activity track with response to oncolytic virotherapy, mRNA vaccines, and poly(I:C). Conceptually, RNase L parallels DNASE1L3 as an inducible endoribonuclease whose deficiency leads to inappropriate ligand accumulation and chronic sensor activation. Just as RNase L handles pathogen-driven RNA, an editing-based mechanism is required to prevent chronic MDA5 engagement by endogenous dsRNA. The enzyme that carries out that role is ADAR1, and its structural biology, deficiency phenotype, and therapeutic targetability constitute the closing entry of this chapter.

### ADAR1: the rna-editing immune gatekeeper

ADAR1 catalyzes A-to-I editing of dsRNA at Alu:Alu inverted repeats, destabilizing endogenous duplexes and removing them from the MDA5-eligible pool, raising the MDA5–MAVS activation threshold [16, 106]. Loss of ADAR1 in mice and humans produces a lethal MDA5-driven type-I interferonopathy. In cancer, this dependency becomes a vulnerability. Tumors with high endogenous dsRNA, particularly BRCA1/2-mutant or genomically unstable tumors, become exquisitely dependent on ADAR1 to silence the MDA5 axis. Pharmacologic or genetic disruption of ADAR1 triggers MDA5–MAVS-mediated tumor cell death and synergizes with PARP inhibitors, immune checkpoint blockade, and DNA-damaging chemotherapy [106–109]. The first reported small-molecule ADAR1 catalytic inhibitor, ADAR1i-124, was identified in 2026 and validated in vivo [19], opening the prospect of clinical translation. ADAR1-dependent dsRNA editing is therefore both a brake on physiologic MDA5 immunity and a therapeutic exposure point that may convert "cold" tumors into MDA5-inflamed states. The ADAR1-substrate-selection model rests on RNA immunoprecipitation and sequencing data from a small number of tumor cell lines and murine xenografts, and human tumor tissue evidence for the specific Alu:Alu editing landscape is still being assembled from pilot single-cell studies.

## Diagnostic and therapeutic translation of DNA/RNA immunity in cancer

The structural and mechanistic groundwork established in the previous two chapters is now being translated into cancer diagnostics and therapeutics at an accelerating pace, and the translation tracks the two-fate biology directly. Tumor-derived DNA and RNA are the substrate on which both the diagnostic and therapeutic pipelines operate: diagnostically, tumor cfDNA fragmentomics reads the nuclease-specific cleavage signatures written onto plasma DNA to detect malignancy at low burden; therapeutically, cancer strategies aim to redirect tumor DNA and RNA out of the immunologically silent fate back into the sensor-engaging fate through inhibition of cytosolic checkpoints, agonism of innate sensors and NET clearance, while diagnostics rely on nuclease-specific fragmentomic signatures written onto plasma DNA. We consolidate diagnostics and therapeutics under a single chapter because they share the same conceptual substrate, namely the tumor-remodelled DNase and RNase repertoire, but they extract opposite information from it: diagnostics quantify the signature, therapeutics dismantle it (Table 2).

**Table 2:** Diagnostic and therapeutic pipeline targeting nucleic-acid-sensing pathways in oncology

Modality	Target/Mechanism	Lead Agent(s)	Indication(s)	Stage	Key Evidence	Citation
DNase replacement + chemotherapy	DNASE1 (NET clearance, restoration of chemo penetration)	Dornase alfa + cisplatin	Refractory germ-cell tumors	Trial-stage	NCT07121322 open-label phase I/II adjuvant; NET-mediated chemoresistance	NCT07121322
DNase replacement (engineered biologic)	DNASE1L3 Fc-fusion (actin-resistant; long half-life)	DNASE1L3-Fc biologic	Hepatocellular carcinoma, colorectal carcinoma	Late preclinical	DNASE1L3 restoration → PANoptosis + αPD-1/sorafenib; Fc-fusion bypasses actin block	[27, 77]
Dual-DNase replacement	Combined DNASE1 + DNASE1L3 substrate breadth (free DNA + microparticle/NET DNA)	Dual-acting DNase biologic	Solid tumors (HCC, CRC) with SLE crossover	Late preclinical	Fc-fusion covers NET + microparticle chromatin; outperforms single enzyme	[77]
STING agonist	STING activation (type-I IFN; cold-to-hot conversion)	ADU-S100, MK-1454, next-generation systemic small-molecule and nanoformulated CDNs	Advanced solid tumors	Trial-stage	First-gen CDN tested in trials; next-gen systemic + nanoformulation to bypass intratumoral-only delivery	[79, 80]
STING agonist (nanomedicine)	Dual-STING-activating micelle programs kinetics + ferroptosis-coupled metal-peroxide nanoparticles	D-SAM micelle; ZnFe₂O₄ and Zn–Cu peroxide nanoparticles	Solid tumors (TNBC; pan-tumor)	Late preclinical (D-SAM)/Early preclinical (nano)	D-SAM extends STING window; metal-peroxide NPs release Zn^2^⁺/Fe^2^⁺ → mtDNA damage + STING	[81–83]
ADAR1 inhibitor	dsRNA/MDA5 synthetic lethality in IFN-stimulated and BRCA1/2-mutant tumors	ADAR1i-124 (first selective small molecule)	BRCA1/2-mutant breast/ovarian; MSI-high; ISG-high solid tumors	Late preclinical	ADAR1 depletion synthetic-lethal with BRCA1/2 (IFN poisoning); ADAR1i-124 first selective A-to-I inhibitor, in-vivo validated	[68]
TREX1 inhibitor	Cytosolic DNA checkpoint relief (re-engagement of cGAS–STING)	Selective TREX1 small-molecule inhibitors ± immune checkpoint blockade	Solid tumors with intrinsic DNA-damage burden	Late preclinical	TREX1 loss derepresses STING + synergizes αPD-1 in breast/PAAD/melanoma; first drug-like inhibitors 2024–25	[56, 58, 59, 78]
RIG-I agonist	dsRNA sensor activation (5′-triphosphate stem-loop RNA)	SLR14 (synthetic 5′-pppRNA stem-loop)	Solid tumors	Early preclinical	Hairpin RIG-I agonist → IFN-β + apoptosis (murine); NP formulation enhances macrophage targeting	—
TLR7/8 agonist	Endosomal ssRNA sensor activation	GY101 (selective TLR7); R848-derived small molecules; nanoparticle-formulated TLR7/8 agonists	Solid tumors	Early preclinical	GY101 → colon/melanoma/breast tumor regression + M2 → M1 repolarization; structure-guided + NP delivery limit cytokine release	[34, 62, 63]
Oncolytic virus + mRNA/RNA nanoparticle vaccine	RNA sensing via dsRNA-rich oncolytic genomes (PVSRIPO) and LNP/RNA-LPA neoantigen vaccines	PVSRIPO; RNA-LPA personalized vaccines; LNP-mRNA platforms	Recurrent glioblastoma, melanoma	Trial-stage	PVSRIPO MDA5 engagement (recurrent GBM trials); RNA-LPA → rapid sensor activation + clinical responses; LNP-mRNA leverages endogenous RNA sensors	—

### Diagnostic platforms built on nuclease-specific signatures

**Fig. 4:**
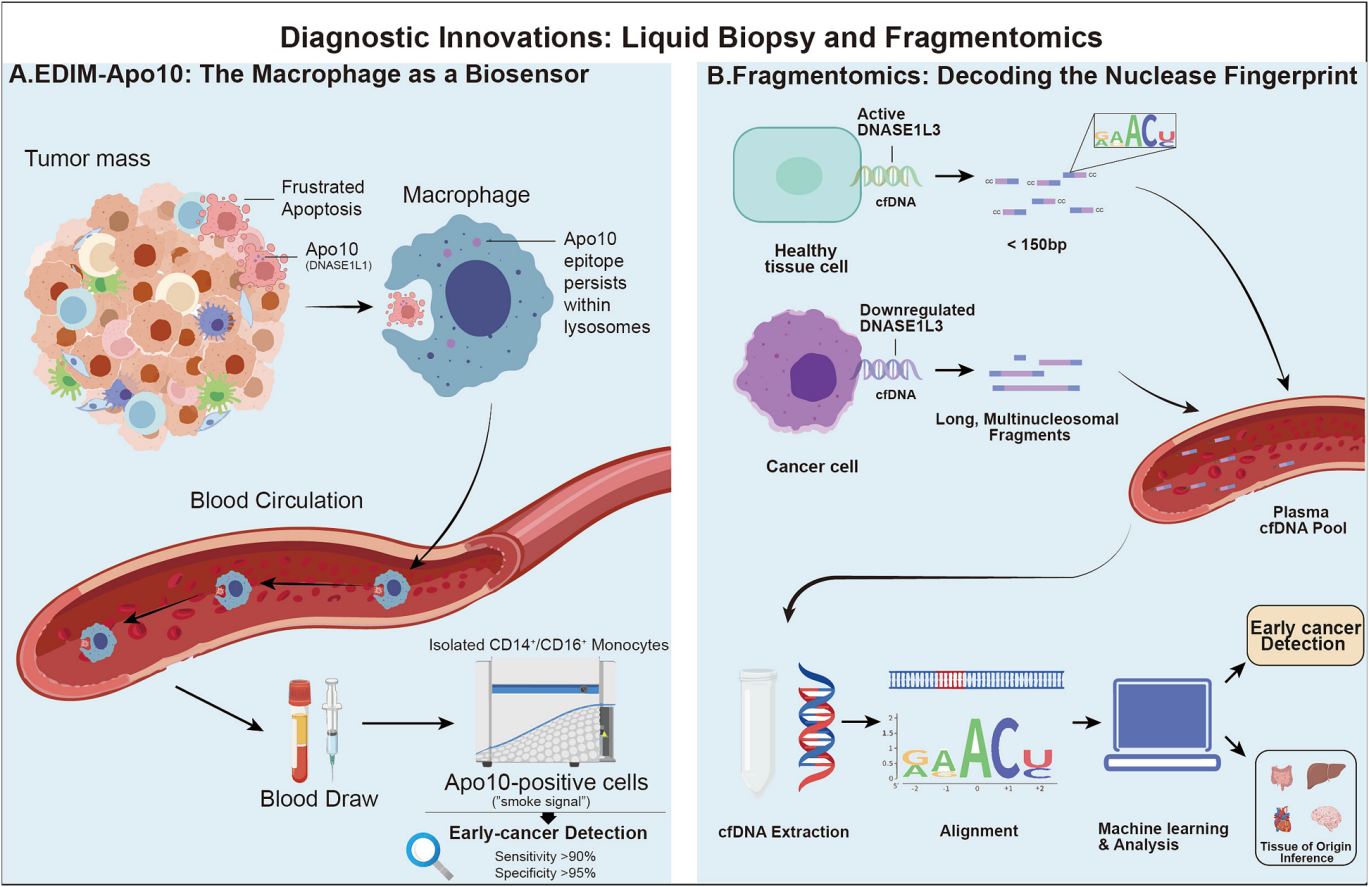
Non-invasive diagnostic platforms exploiting DNASE-specific cleavage signatures and cellular epitope accumulation. **A** The epitope detection in monocytes (EDIM) platform. Malignant cells undergoing "frustrated apoptosis" fail to execute complete chromatin degradation, resulting in the abnormal nuclear accumulation of DNASE1L1 (defined as the Apo10 epitope). Circulating CD14⁺ monocytes/macrophages phagocytose these tumor-derived apoptotic bodies, leading to the formation of stable Apo10 aggregates that persist within their lysosomes. By isolating these circulating phagocytes from a routine blood draw, intracellular Apo10 acts as a concentrated, systemic biosensor (a "smoke signal"), enabling the detection of early-stage malignancies with exceptionally high sensitivity and specificity, circumventing the dilution limitations of conventional soluble biomarkers. **B** Cell-free DNA (cfDNA) fragmentomics and nuclease fingerprinting. In healthy physiological states, active extracellular DNASE1L3 orchestrates the precise cleavage of chromatin, generating predominantly short cfDNA fragments (< 150 bp) characterized by specific "CC" dinucleotide end-motifs and jagged termini. Conversely, in malignancies associated with DNASE1L3 downregulation (e.g., HCC, CRC), this physiological nuclease fingerprint is profoundly altered, resulting in an increased proportion of longer, multinucleosomal cfDNA fragments with aberrant end-motif frequencies. Following cfDNA extraction and whole-genome alignment, advanced machine-learning algorithms decode these distinct fragmentation profiles to facilitate early cancer detection and infer the tumor's tissue of origin

Nucleic acid-related diagnostic approaches are illustrated in Fig. 4. Two conceptually distinct platforms have emerged from the nuclease biology developed above, and they interrogate complementary layers of the same tumor signal. The first reads a cellular consequence of frustrated apoptosis captured inside circulating macrophages (EDIM–Apo10), whereas the second reads the enzymatic cleavage fingerprint written directly onto cell-free DNA (fragmentomics). We consider each in turn and then position them as orthogonal, mutually reinforcing readouts rather than competing assays.

#### EDIM–Apo10: The macrophage as a biosensor

EDIM uses circulating monocytes as carriers of tumor-derived molecular signatures [110]. Macrophages phagocytose tumor cells with frustrated apoptosis and accumulate the Apo10 epitope in their lysosomes. Routine venipuncture isolation followed by intracellular staining for Apo10 and the anaerobic metabolism marker TKTL1 achieves high sensitivity above 90% and specificity above 95% for breast, prostate, pancreatic, and oral malignancies [29]. Active concentration by macrophages mitigates dilutional limitations of soluble biomarkers and may signal occult malignancy before radiographic detection. EDIM operates through a cellular biosensor logic; fragmentomics, by contrast, reads the enzymatic fingerprint directly from cell-free DNA, and the two platforms therefore constitute complementary rather than competing modalities.

#### Fragmentomics: decoding the nuclease fingerprint

Fragmentomics analyzes plasma cfDNA cleavage patterns [9]. DNASE1L3 preferentially cleaves at CC dinucleotides, producing short fragments below 150 bp with jagged ends [25]. In HCC, CRC, and other DNASE1L3-low malignancies, the cfDNA landscape shifts toward reduced CC end-motifs and longer multinucleosomal fragments [111–114]. In a 2025 multi-cancer validation involving 3,021 patients and 3,370 controls, AUC values exceeded 0.98 for overall cancer detection, with 87.4% sensitivity at 97.8% specificity across 13 cancer types and 82.4% tissue-of-origin accuracy [18]. Subsequent 2025–2026 work has extended this principle. The CANSCAN platform applies AI-driven multi-modal fragmentomics to large prospective cohorts. ELSM integrates fragmentomics with shallow whole-genome sequencing [106]. CEliver achieves 98% sensitivity for early HCC detection [115]. FLARE captures fragment-level methylation via nanopore sequencing [116–120]. The convergence of these tools shows that the nuclease fingerprint is a robust signal across sequencing platforms. Mechanistically, DNASE1L3 loss in HCC drives macrophage polarization through the NLRP3–GSDMD axis and converts apoptotic death into a PANoptosis-skewed program that amplifies inflammatory cytokine release [121, 122], so DNASE1L3 expression is both a fragmentomic determinant and a prognostic biomarker [123].

### Therapeutic strategies on the DNA side

**Fig. 5:**
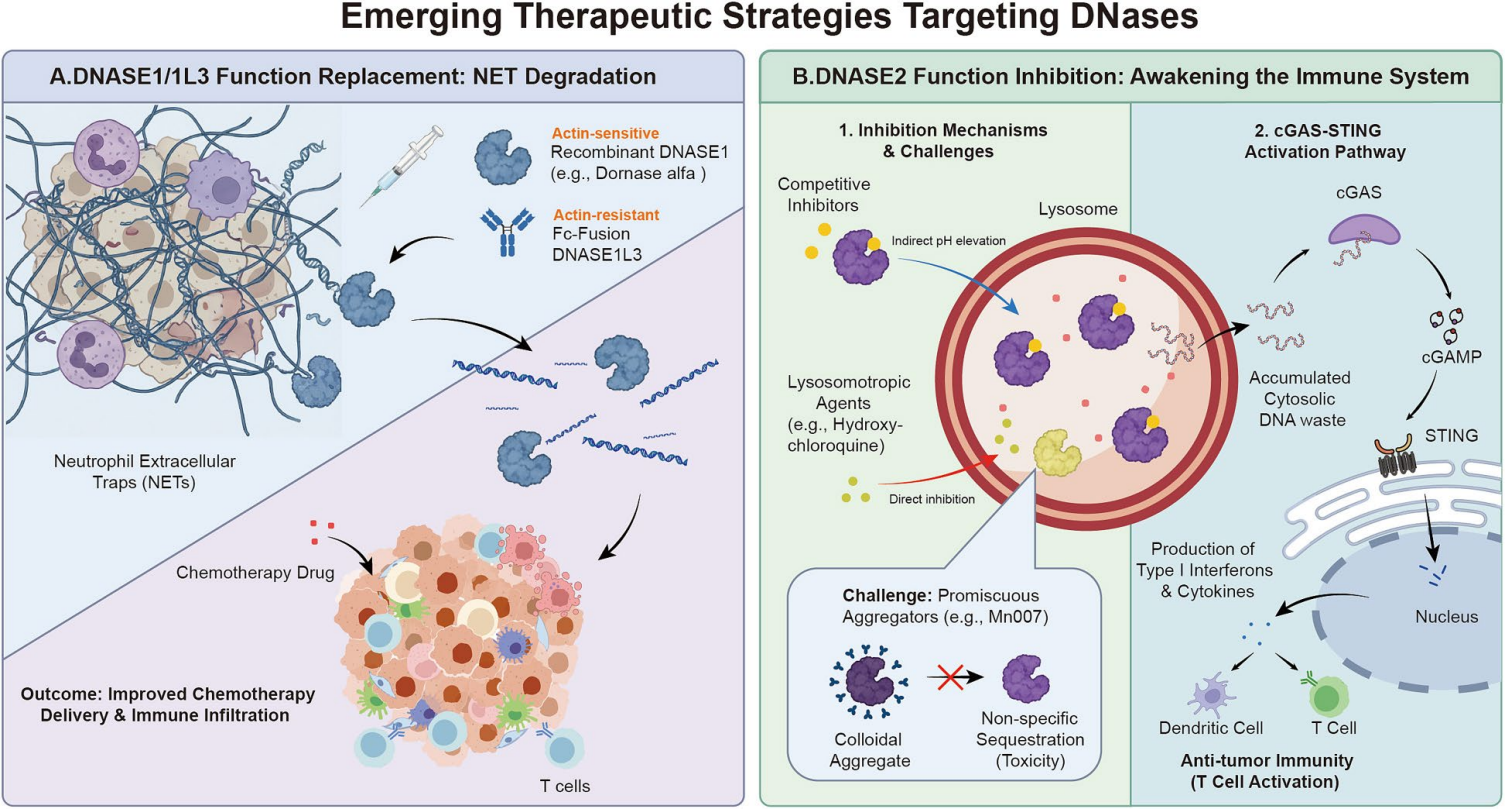
Emerging therapeutic strategies targeting the DNases family to rewire the immune-metabolic tumor microenvironment. **A** Function replacement strategies: Dismantling the physical metastatic niche. Therapeutic restoration of extracellular nucleolytic capacity aims to clear Neutrophil Extracellular Traps (NETs) that physically shield solid tumors. Systemic administration of recombinant wild-type DNASE1 (e.g., Dornase alfa) or next-generation engineered, actin-resistant Fc-fused DNASE1L3 actively digests these dense extracellular chromatin webs. This NET degradation disrupts the metastasis-supporting scaffold, restores microvascular patency, significantly enhances the intra-tumoral delivery of chemotherapeutic agents, and facilitates the robust infiltration of cytotoxic T cells, thereby overcoming immune exclusion. **B** Function inhibition strategies: Awakening immune sensing via DNASE2 blockade. Targeted pharmacological inhibition of lysosomal DNASE2 is designed to reignite the suppressed cGAS-STING alarm pathway in immunologically "cold" tumors. Left subpanel: Current modalities range from non-specific lysosomotropic agents (e.g., hydroxychloroquine) that indirectly compromise enzyme function by elevating lysosomal pH, to the rational design of active-site competitive inhibitors. A significant medicinal chemistry challenge remains the exclusion of "promiscuous aggregators" (e.g., Mn007), which act via non-specific colloidal sequestration rather than true active-site binding, often leading to unacceptable systemic toxicity. Right subpanel: Successful and specific DNASE2 inhibition forces the intra-lysosomal accumulation of undigested DNA debris, which ultimately overflows into the cytosol. This accumulated cytosolic DNA waste potently triggers the canonical cGAS-cGAMP-STING axis, culminating in the robust production of Type I interferons and pro-inflammatory cytokines. This molecular cascade effectively reprograms the tumor microenvironment by activating dendritic cells and promoting cross-priming, thereby mounting a durable anti-tumor T cell immune response

DNA-related tumor therapies are illustrated in Fig. 5. Therapeutic strategy on the DNA side follows directly from the two-fate logic: where a protective nuclease activity is lost or actin-inhibited, the aim is to restore it, and where a cytosolic nuclease actively silences sensing, the aim is to remove or inhibit it. The subsections below trace this logic across three intervention classes, extracellular enzyme replacement (DNASE1 and engineered DNASE1L3), cell-autonomous checkpoint inhibition (TREX1), and direct sensor agonism (STING) which together convert immunologically silent tumor DNA back into a sensor-engaging ligand.

#### Function replacement: Dornase Alfa and engineered DNASE1L3

Dornase alfa, a recombinant DNASE1 approved for cystic fibrosis, is being investigated in oncology against NET-rich microenvironments. Preclinical studies show that systemic DNASE1 dissolves NETs, reduces hepatic and pulmonary metastatic seeding, and improves response to immune checkpoint inhibitors. NCT07121322 evaluates dornase alfa in refractory germ cell tumors, where NET degradation is expected to improve pulmonary function and facilitate cisplatin delivery. Wild-type DNASE1 nevertheless remains constrained by actin inhibition in the actin-rich tumor core [37]. Attention has therefore shifted to DNASE1L3, which is intrinsically actin-resistant and accesses membrane-protected DNA, and to engineered Fc-fused chimeras with extended half-life. In HCC and colon cancer models, restored DNASE1L3 activity triggers PANoptosis, dismantles NETs, and increases sensitivity to anti–PD-1 and sorafenib. The recent dual-acting DNASE1/DNASE1L3 biologic conferring both actin resistance and microparticle access establishes a preclinically compelling template that bridges oncology and autoimmunity and that awaits first-in-human validation, particularly with respect to pharmacokinetics, immunogenicity, and dosing schedule in tumor-bearing hosts [124]. The mirror-image application of the same biologic in DNASE1L3-deficient lupus is developed in the non-tumor chapter. Enzyme replacement operates entirely in the extracellular space, adding a missing nucleolytic activity to the tumor microenvironment. The mirror-image intervention removes an unwanted nucleolytic activity from inside the tumor cell, converting a cytosolic nuclease into a druggable checkpoint that clears itself out of the way of cGAS.

#### TREX1 as a tumor-cell-autonomous cytosolic checkpoint

TREX1 has emerged as one of the most actionable cytosolic DNA-arm targets. Genetic or pharmacological TREX1 disruption in tumor cells permits accumulation of cytosolic DNA from replication stress and irradiation, driving cGAS–STING activation, type I IFN, and dendritic cell cross-priming [13, 14]. In murine breast, pancreatic, and melanoma models, TREX1 loss synergizes with checkpoint blockade to produce durable regression and protective memory, and conditional intratumoral TREX1 induction by small molecules likewise converts non-inflamed tumors into responders [15, 73, 125]. The first selective TREX1 small-molecule inhibitors with drug-like properties were disclosed in 2024–2025 and are now in late preclinical development. Because TREX1 inhibition activates the same downstream pathway as STING agonism but operates cell-autonomously within tumor cells, the two strategies may be synergistic. TREX1 disruption and STING agonism converge on the same downstream axis, and combining the two, together with the delivery chemistry that determines whether either reaches the tumor without triggering systemic interferon toxicity, defines the pharmacological frontier where the DNA-arm therapeutic argument now sits.

#### STING agonists and the nanomedicine frontier

STING agonists aim to convert cold tumors into hot ones by mimicking cGAMP. First-generation cyclic dinucleotides such as ADU-S100 and MK-1454 showed proof of concept but were limited by poor pharmacokinetics, intratumoral-only administration, and modest single-agent activity [126, 127]. The next generation pursues three complementary strategies. The first is systemic small-molecule agonists with improved drug-like properties in phase I/II combinations with anti–PD-1/PD-L1. The second is nanomedicine delivery, illustrated by the dual-STING-activating micelle D-SAM that dynamically programs STING kinetics and extends the immunotherapeutic window [128]; ZnFe₂O₄ nanosystems that release Zn^2^⁺ and Fe^2^⁺ to generate mitochondrial DNA damage and downstream cGAS–STING activation while delivering MRI imaging [129]; and bimetallic Zn–Cu peroxide nanoparticles that induce ferroptosis and cuproptosis concurrent with STING activation [130]. The cellular-effector axis, still at the earliest stage of translation, is being tackled by STING-coupled cellular therapies such as STING-armed CAR-T, which are entering preclinical proof-of-concept in TNBC. Across all three axes, the narrow first-generation therapeutic window is being addressed through chemistry, materials, and delivery rather than ligand optimization alone. The DNA-arm portfolio outlined above, replacement of extracellular DNase, inhibition of the cytosolic checkpoint TREX1, and agonism of STING, has a structural mirror on the RNA arm, and it is that mirror-arm portfolio that occupies the discussion that follows.

### Therapeutic strategies on the RNA side

Pharmacological STING activation defines one pole of the cytosolic DNA-arm strategy; its conceptual mirror on the RNA arm is engagement of the RIG-I/MDA5/TLR7/TLR8 sensor network with synthetic or therapeutic RNA ligands. The premise is identical to the DNA-arm logic, namely that the cytosolic and endosomal sensing machinery is intact in most solid tumors but is starved of qualifying ligand because tumor-cell ADAR1 editing and other guard rails suppress immunogenic dsRNA accumulation [16, 102]. Pharmacological interventions on the RNA arm therefore partition into three operating modes that parallel the DNA-arm portfolio of dornase alfa replacement, TREX1 inhibition, and STING agonism: direct agonism of RNA sensors with engineered ligands, removal of the ADAR1 editing brake to release endogenous dsRNA, and selective interference with RNase H2 to deliberately permit cytosolic RNA:DNA hybrid accumulation. The clinical maturity of these three modes varies, but each has at least one validated chemical probe and a defined cancer indication.

#### RIG-I/MDA5 and TLR7/TLR8 agonism

Direct sensor agonism on the RNA arm draws on a chemistry base that small-molecule cGAMP analogs cannot easily access on the DNA arm. Selective TLR7/TLR8 agonists exploit endosomal localization and constitutive expression on plasmacytoid dendritic cells and B cells, producing tumor-infiltrate-rich IFN bursts with manageable systemic toxicity profiles in early-phase oncology settings. A series of next-generation TLR7-selective agonists, including GY101, have demonstrated single-agent activity in murine colorectal models through dendritic cell licensing and CD8 effector reprogramming [103], and a broader pipeline of vaccine-conjugated and ADC-formatted TLR7/TLR8 agonists is now in combination trials with checkpoint blockade [104, 105]. Structure-guided design of endosomal TLR ligands continues to address the principal liability of this class, namely the trade-off between potency and systemic interferon toxicity [105]. The RIG-I and MDA5 arms remain less clinically mature than TLR7/TLR8, but recent ADAR1 work has provided a route to engage them indirectly through removal of the ADAR1-imposed editing brake.

#### ADAR1 disruption for synthetic lethality

Tumor cells with elevated endogenous dsRNA load become dependent on ADAR1 catalytic editing to prevent MDA5 self-recognition, and this dependency is the actionable lever for an entire new therapeutic class. Mechanistic refinement of the ADAR1 dependency in 2024–2025 identified the relevant immunogenic substrate as a defined subset of Alu:Alu inverted-repeat duplexes whose editing efficiency dictates whether MDA5 is triggered or silenced [106], and the dependency was most pronounced in lung adenocarcinoma, where ADAR1 inhibition restored IFN signaling and sensitized tumors to immune checkpoint blockade [108, 109]. The mRNA processing context that determines ADAR1 substrate availability has also been clarified, providing a basis for patient selection beyond bulk ADAR1 expression [107]. These observations established a clear translational hypothesis, and the first selective ADAR1 catalytic inhibitor with reported in vivo activity, ADAR1i-124, validated the hypothesis by demonstrating MDA5-dependent antitumor efficacy in xenograft models [19]. The same logic of removing a guard rail to permit cytosolic nucleic acid accumulation can be applied to a parallel RNA-arm restraint, the RNase H2 complex. The same logic of removing a guard rail on cytosolic nucleic-acid sensing extends to RNase H2, which we address next.

#### RNase H2 as an emerging replication-stress target

RNase H2 occupies a position analogous to TREX1 on the RNA arm in that it suppresses cytosolic nucleic acid accumulation by resolving RNA:DNA hybrids at sites of replication-transcription conflict, and tumor cells appear to upregulate this resolution capacity to tolerate elevated transcriptional stress [30]. Genetic depletion of RNase H2 in TNBC and other replication-stress-prone tumors elevates cytosolic RNA:DNA hybrid accumulation, triggers cGAS-STING activation, and produces marked synergy with PARP and platinum agents [50]. Selective small-molecule RNase H2 inhibitors are not yet clinical, but the mechanistic case is now strong enough that medicinal chemistry programs targeting this complex are credible. RNA-arm therapy as a whole, namely sensor agonism plus ADAR1 inhibition plus RNase H2 inhibition, therefore mirrors the DNA-arm portfolio that runs from STING agonism through TREX1 inhibition: each strategy lifts a different physiological restraint on cytosolic nucleic acid sensing. This symmetry brings the same translational problem into sharp focus on both arms, namely that nuclease inhibition unlocks immunogenic nucleic acid pools the host normally clears for good reason, and the disease-modifying biology of the targeted nuclease cannot be ignored once therapeutic intervention is proposed. The same nuclease-inhibition principle recurs on the DNA arm, where it is most conspicuously realized and most conspicuously problematic in DNASE2, whose translational profile crystallises the dilemma of exploiting a physiologically essential nuclease for oncology and demands the dedicated risk analysis that closes the DNA-arm therapeutic discussion.

### The two-sided DNASE2 problem

DNASE2 inhibition is the most direct route to forcing cytosolic DNA overflow in tumor-associated macrophages and the most thoroughly cautionary illustration of the same nuclease-inhibition principle that motivates TREX1 and RNase H2 work. On the chemistry side, the published high-throughput hits including the lead compound Mn007 act primarily through colloidal aggregation rather than active-site engagement, sequestering DNASE2 nonspecifically in physiologic buffers; rigorous re-evaluation has placed this entire compound series in the promiscuous-aggregator category and no co-crystal of human DNASE2 with a bona fide active-site inhibitor has been deposited [75]. Lysosomotropic agents such as hydroxychloroquine elevate endolysosomal pH and indirectly attenuate DNASE2 activity, but their pharmacology is broad and they are unsuitable as selective probes [131]. The field has therefore not converged on a clean tool compound, which means that every claim of DNASE2-pathway dependence in tumor models drawn from these chemical probes warrants confirmation through orthogonal genetic perturbation.

The chemistry gap is the lesser of two problems. The same biology that motivates DNASE2 inhibition, namely intralysosomal DNA debris overflow into cytosolic cGAS-STING, is itself a well-characterized driver of systemic immunopathology. Murine and human evidence shows that DNASE2 loss-of-function produces severe fetal-liver erythroid arrest, chronic macrophage activation, type I interferonopathy, and polyarthritis-like inflammation, and the OAS-RNase L axis discussed above among the RNA sensors contributes to the postnatal macrophage phenotype [12, 45]. Systemic DNASE2 inhibition in adult cancer patients would therefore carry a non-trivial risk of macrophage activation syndrome, hemolytic anemia, and ICI-independent autoinflammatory toxicity, with the toxicity profile likely most pronounced under concomitant checkpoint blockade. Tractable mitigations exist, including tumor-targeted delivery through mannose-receptor-conjugated nanocarriers, short-half-life reversible inhibitors with intermittent dosing schedules, and the use of tumor-cell-autonomous alternatives such as TREX1 inhibition that bypass macrophage DNASE2 biology entirely [124]. The same translational concern, namely that engineering around a nuclease whose physiological function is the prevention of autoimmunity demands tight therapeutic-window control, applies with equal force on the opposite side of the DNA-arm intervention, namely the use of recombinant DNASE1L3 as a replacement biologic.

### The immunogenicity risk of DNASE1L3 replacement

DNASE1L3 occupies a particular position among DNA-arm therapeutic targets because it is simultaneously a high-priority replacement candidate, validated by oncology and autoimmunity models alike, and one of the principal autoantigens in human systemic lupus erythematosus [132]. Replacement biologics built on this enzyme therefore carry a class-specific immunogenicity ceiling that small-molecule TREX1 inhibitors and recombinant DNASE1 dornase alfa do not. Anti-drug antibody formation in patients with subclinical autoreactivity, particularly those with elevated baseline anti-DNASE1L3 titers, can both neutralize the recombinant enzyme and amplify the underlying autoimmune phenotype, and the Fc fusion strategies used to extend serum half-life add B-cell-receptor-engaging epitopes that may compound this risk. The dual-acting DNASE1/DNASE1L3 chimera reported by Stabach and colleagues addresses part of the problem by using the catalytic domain of DNASE1L3 within a DNASE1 scaffold that retains actin-resistance and microparticle access without presenting the full DNASE1L3 epitope set [124], and the engineering principle, namely selective transfer of biochemical activity onto an immunologically tractable backbone, offers a conceptually compelling but preclinically limited path through this constraint, and its clinical viability will hinge on whether the reduced epitope set indeed lowers anti-drug antibody incidence in patients with baseline anti-DNASE1L3 autoreactivity.

The clinical implications follow directly from this immunological landscape. Patients with documented anti-DNASE1L3 autoantibodies represent a contraindicated subgroup for any DNASE1L3-derived biologic, and biomarker-driven exclusion at trial entry should become standard practice rather than a post-hoc safety amendment. For tumors in which the same biology motivates a DNASE1L3-replacement strategy, namely those characterized by NET-rich microenvironments coupled with elevated chromatin microparticle burden such as HCC, CRC, and pancreatic adenocarcinoma [121], the immunogenicity calculus is more favorable than in established autoimmunity but still demands sustained monitoring during chronic dosing. An alternative DNA-arm strategy in immunogenicity-restricted patients is intracellular TREX1 inhibition with small molecules, where the lower molecular-weight chemical matter does not produce neutralizing antibody responses and the cell-autonomous mechanism does not depend on sustained serum enzyme exposure [13]. The decisive observation is that DNASE1L3 itself recurs across both the cancer immune-evasion landscape developed above and the autoimmune-clearance-failure landscape of lupus and the broader interferonopathy spectrum, but the direction of therapeutic intervention reverses across that boundary, and the same enzyme that requires augmentation in oncology is the principal autoantigen and the principal replacement target in lupus. Because DNASE1L3 recurs in opposite therapeutic direction in lupus, the natural continuation of this chapter is the non-tumor setting, where the same enzymes are replaced rather than inhibited.

## Diagnostic and therapeutic translation in non-tumor diseases

**Fig. 6:**
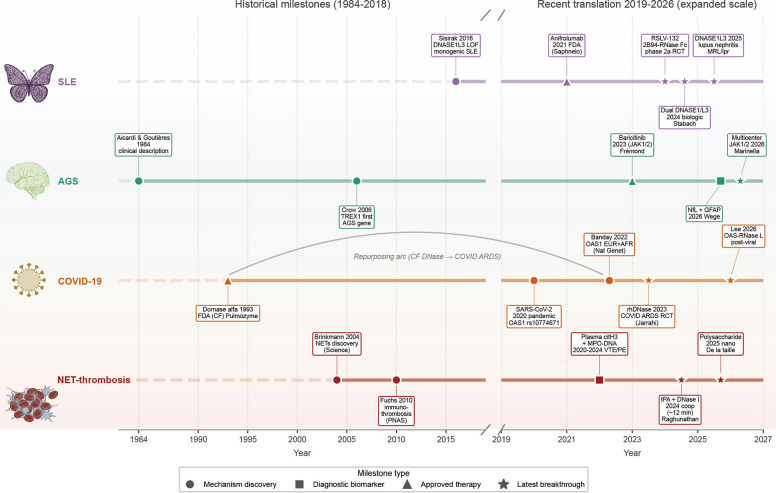
Clinical and late-preclinical translation of DNA- and RNA-arm interventions across four non-tumor diseases. This timeline (1984–2026) uses a broken scale to detail milestones in SLE, AGS, COVID-19/ARDS, and NET-driven thrombosis, categorized by mechanism (●), biomarkers (■), approved drugs (▲), and breakthroughs (★). Key events include *DNASE1L3* discoveries and Anifrolumab approval in SLE; *TREX1* identification and JAK inhibitor trials in AGS; the repurposing of Dornase alfa and *OAS1* studies in COVID-19; and the evolution from NET discovery to nanoparticle thrombolysis. The figure highlights the 1993 Dornase alfa approval as a platform anchor, illustrating varying translation tempos and the recent shift toward dual-acting biologics and engineered nucleases

The sensing–clearance equilibrium developed structurally and mechanistically in the preceding chapters fails in disease contexts well beyond cancer, and we now examine the settings in which DNA- or RNA-arm dysregulation both drives substantial unmet clinical need and has produced the most concrete translational progress. Systemic lupus erythematosus is treated as the prototype disease of DNASE1L3 deficiency, in which loss of extracellular chromatin clearance couples directly to type I interferon signalling and to the C1q–DNASE1 crosstalk developed above; Aicardi–Goutières syndrome is treated as the interferonopathy that maps the trinity onto human disease through TREX1, RNase H2 and RNase T2 mutations and, more recently, through JAK1/2 inhibition as effective standard of care; COVID-19 is treated as the acute infection in which the OAS–RNase L axis, DNase-mediated NET clearance and their pharmacologic manipulation converge; and NET-driven cardiovascular, thrombotic and septic disease is treated as the chronic non-tumour setting in which the DNASE1/DNASE1L3 balance and the complement crosstalk drive substantial population-level morbidity. Across these settings the same enzymes that promote tumour immune evasion when lost become therapeutic agents when restored, and the same sensors that drive antitumour immunity when activated become therapeutic targets for blockade when chronically engaged; a synthesis chapter then extracts the cross-disease lessons and the therapeutic-window problem that this directional duality creates. This is the directional duality, replacement where loss drives disease, inhibition where gain drives disease, that constitutes the connective tissue of the chapter and is summarised in Fig. 6 and in the corresponding pipeline in Table 3.

**Table 3:** Clinical and late-preclinical pipeline targeting nucleic-acid-sensing pathways in non-malignant disease

Modality	Target/Mechanism	Lead Agent(s)	Indication(s)	Stage	Key Evidence	Citation
DNase replacement (inhaled)	DNASE1 (mucus DNA hydrolysis; NET clearance)	Dornase alfa (Pulmozyme)	Cystic fibrosis	Approved (FDA 1993)	Only nucleic-acid–targeting biologic approved as long-term maintenance; reduces exacerbations, slows FEV₁ decline	[4]
DNase replacement (repurposed IV)	DNASE1 (NET clearance in ARDS/cytokine storm)	rhDNase I, IV infusion	COVID-19 ARDS	Trial-stage	Pilot trials ↓ NET markers (cfDNA, MPO–DNA) + trend ↑ oxygenation in severe COVID-19	[108]
DNase + thrombolytic co-delivery	DNASE1 + tPA (NET dissolution + fibrinolysis)	Nanoparticle-formulated tPA + DNase I	Acute ischemic stroke	Late preclinical	Co-delivery NP clot lysis ~12 min in murine MCAO; outperforms either agent alone in NET-rich thrombi	[117, 118]
DNase replacement (engineered biologic)	DNASE1L3 Fc-fusion or dual DNASE1/DNASE1L3 (microparticle DNA + NET clearance)	DNASE1L3-Fc; dual-acting DNase biologic	Systemic lupus erythematosus (especially with hereditary DNASE1L3 deficiency)	Late preclinical	Long–t½ Fc-fusion restores serum DNase activity in Dnase1l3⁻/⁻ mice ↓ anti-dsDNA; dual biologic broadens substrate	[77]
RNase replacement (Fc-fusion)	Extracellular RNA/immune-complex RNA hydrolysis	RSLV-132	Systemic lupus erythematosus	Trial-stage (Phase 2a)	Phase 2a SLE: favorable safety, target engagement (↓ immune-complex RNA), exploratory fatigue improvement	[88]
JAK1/2 inhibitor	Type-I IFN signaling blockade (downstream of cytosolic-nucleic-acid sensors)	Baricitinib	Aicardi–Goutières syndrome (off-label)	Trial-stage/standard of care	Multi-center observational ↓ neurological progression + ISG normalization; emerging first-line	[99, 100]
JAK1/2 inhibitor	Type-I IFN signaling blockade	Ruxolitinib	Aicardi–Goutières syndrome	Trial-stage	Multi-center retrospective vs. baricitinib: similar extra-neurological gains, limited/heterogeneous neuro benefit, favorable safety	[101, 102]
DNase replacement (NET-focused)	DNASE1 (NET clearance in lupus nephritis)	Recombinant DNase I (preclinical)	Lupus nephritis (MRL/lpr preclinical)	Late preclinical	Systemic DNase I in MRL/lpr ↓ renal NETs (MPO, citH3), ↓ IL-1β/TNF-α/Kim1, ↑ renal pathology; complements anifrolumab on IFN axis	[87]

### Systemic lupus erythematosus: the prototype disease of DNASE1L3 deficiency

Systemic lupus erythematosus (SLE) is the prototype disease of nucleic acid clearance failure on the DNA arm. Biallelic loss-of-function variants in DNASE1L3 cause a familial hypocomplementemic urticarial vasculitis–like SLE characterized by early onset, severe glomerulonephritis, and very high titers of antinuclear antibodies, particularly anti-chromatin antibodies [132]. Even in patients without monogenic DNASE1L3 loss, reduced serum DNASE1L3 activity and elevated anti-DNASE1L3 autoantibodies are observed in a subset of sporadic adult SLE, and these features correlate with disease activity and renal involvement [133]. The mechanistic chain from DNASE1L3 deficiency through accumulation of chromatin-laden microparticles to chronic cGAS–STING activation and pathogenic anti-DNA antibody production has been recapitulated in murine Dnase1l3-null models and, more recently, in patient-derived monocyte and dendritic cell systems [134]. Murine MRL/lpr lupus is similarly improved by exogenous DNase I, with reductions in NET formation, renal inflammation, and proteinuria [135]. SLE illustrates the loss-of-function DNase phenotype in its most common polygenic form, driven predominantly by DNASE1L3 activity failure and NET clearance failure at the level of the tissue. The same failure mode, telescoped into a strictly Mendelian and pediatric-onset genetic architecture, produces the interferonopathy of Aicardi–Goutières syndrome, where biallelic loss of a single intracellular nuclease is causally sufficient for disease.

Translational implications follow directly. Replacement therapy with engineered actin-resistant DNASE1, or with the dual-acting DNASE1/DNASE1L3 biologic reported in 2024, has produced striking efficacy in genetic and induced lupus mouse models, including reductions in autoantibody titers, glomerular immune complex deposition, and proteinuria, supporting first-in-human evaluation [124]. Biomarker development based on cfDNA fragmentomics, DNASE1L3 enzyme activity assays, and circulating eccDNA signatures is being adapted to SLE diagnosis and disease-activity monitoring, with several large prospective cohorts now in follow-up [135]. RNA-arm therapy in SLE is illustrated by RSLV-132, a recombinant RNase Fc-fusion biologic designed to digest immunostimulatory RNA in immune complexes. A phase 2a randomized clinical trial in active SLE demonstrated a trend toward clinical improvement in patients with more active systemic disease, providing the first human evidence that RNase replacement is therapeutically feasible and supporting expansion of the approach across nucleic-acid–driven autoimmunity [20]. The RSLV-132 result is particularly important for the present review because it establishes that the conceptual symmetry between DNase replacement on the DNA arm and RNase replacement on the RNA arm is not only mechanistic but clinical. Finally, the cGAS–STING inhibitor pipeline for SLE and lupus nephritis has matured rapidly in 2024–2026 with the entry of selective small-molecule cGAS and STING inhibitors into late preclinical development and early clinical evaluation [136–139], and the broader pathway biology has been recently reviewed in this journal [140].

### Aicardi–Goutières syndrome: a TREX1, RNase H2, and RNase T2 interferonopathy

Aicardi–Goutières syndrome (AGS) is a genetic type I interferonopathy caused by loss-of-function variants in nine genes, namely TREX1, RNASEH2A, RNASEH2B, RNASEH2C, SAMHD1, ADAR1, IFIH1 encoding MDA5, LSM11, and RNU7-1. The shared mechanism is chronic activation of cytosolic nucleic acid sensing, operating through cGAS–STING in patients carrying TREX1, SAMHD1, and RNASEH2 variants, and through MDA5–MAVS in patients carrying ADAR1 and gain-of-function MDA5 variants, producing sustained type I interferon signatures, encephalopathy, basal ganglia calcifications, and skin and systemic inflammation [141–143]. The disease provides direct human evidence that loss of nucleic acid clearance produces disease, with TREX1 patients phenocopying Trex1-null mice and RNase H2 patients accumulating ribonucleotide-incorporated genomic DNA [144, 145]. RNASET2-deficient patients additionally accumulate undegraded ribosomal RNA in lysosomes, producing a phenotypically overlapping leukoencephalopathy.

The principal therapeutic advance in AGS over the past five years has been the clinical use of JAK1/2 inhibitors to interrupt the interferon receptor signaling cascade downstream of the persistent IFN output. Baricitinib has produced measurable improvements in skin lesions, intracranial inflammation, and developmental progression in multicenter cohort studies [21, 146]. A 2026 controlled comparison of JAK1/2 inhibition with conventional immunosuppression in a national AGS cohort further established baricitinib as the current standard of care, with manageable infectious complications and the principal residual concern being central nervous system bioavailability [22]. The broader emerging-drug landscape for AGS is reviewed in [147], and biomarkers based on neurofilament light chain and glial fibrillary acidic protein have been validated for monitoring CNS disease activity and treatment response [148]. Notable limitations include incomplete CNS penetration of JAK inhibitors, persistent disease activity in patients with severe brain involvement, and reports of CNS infectious sequelae that have prompted ongoing risk–benefit reassessment [149]. Conceptually, AGS demonstrates the therapeutic mirror of cancer. Where tumors benefit from cytosolic DNase blockade to convert "cold" to "hot," AGS patients benefit from upstream cGAS–STING blockade or downstream JAK inhibition to convert chronic interferonopathy to a manageable inflammatory tone. The monogenic interferonopathy of AGS provides direct human evidence that intracellular nuclease loss is causally sufficient for disease. The same sensing–clearance imbalance can also be produced transiently, not by loss of an enzyme but by a viral RNA ligand that overwhelms an otherwise competent clearance axis, a configuration realised at pandemic scale by SARS-CoV-2.

### COVID-19 and the OAS–RNase L–DNase triple axis in antiviral immunity

The COVID-19 pandemic provided a large-scale natural experiment in nucleic acid–driven immunopathology and confirmed the OAS–RNase L axis as both protective in early infection and pathologic in severe and post-acute disease. The 5'-untranslated region of SARS-CoV-2 is an unusually strong OAS1 activator, and biallelic protective haplotypes at OAS1 are associated with significantly reduced risk of severe COVID-19 hospitalization, independently confirming the in vivo role of OAS1 as a host restriction factor [150–152]. The downstream RNase L cleavage product re-engages RIG-I, propagating the antiviral state to neighboring uninfected cells via paracrine 2-5A signaling, and human genetic studies have used this OAS–RNase L "experiment of nature" to refine the conditions under which the pathway is protective versus damaging.

Severe COVID-19 also features extensive NET formation in the pulmonary microvasculature, immunothrombosis, and acute respiratory distress syndrome. A randomized clinical trial of intravenous recombinant human DNase I in COVID-19 ARDS demonstrated reductions in cfDNA, citrullinated histone H3, and inflammatory markers, with a trend toward improved respiratory parameters in the treatment arm; the trial established the safety of DNase repurposing in this clinical context and motivated subsequent trials in non-COVID ARDS [153]. The broader innate sensing context of severe COVID-19 has been reviewed in [154], with continued interest in chronic cGAS–STING activation as a mechanistic candidate for long COVID. The COVID-19 experience reinforces the therapeutic principle that the DNase replacement strategy originally developed for cystic fibrosis as dornase alfa is portable to other NET-rich settings, while the OAS–RNase L pathway represents a host-genetics-informed druggable axis on the RNA side. Antiviral immunopathology exposes how acquired NET clearance failure amplifies acute lung injury, and the same NET-driven pathology, in a distinct clinical setting and without a defined viral trigger, is what drives the chronic thrombo-inflammatory cascade of cardiovascular disease.

### NET-driven cardiovascular, thrombotic, and septic disease

The NET biology developed in the tumor context is even more clinically advanced in cardiovascular and thrombotic disease. Elevated circulating cfDNA, citrullinated histone H3, and myeloperoxidase–DNA complexes, namely the canonical NETosis markers, are independent predictors of mortality in acute pulmonary embolism, ischemic stroke, sepsis-induced coagulopathy, and atherothrombosis [155–158]. The mechanistic connection is direct. NET chromatin provides a scaffold for fibrin polymerization, sequesters platelets via histones, and exposes tissue factor through neutrophil elastase–mediated cleavage of plasma proteins, producing a uniquely thrombogenic immunothrombotic milieu that is resistant to conventional anticoagulation [159, 160].

Therapeutic translation in this domain centers on DNase-augmented thrombolysis. In acute ischemic stroke, NET-rich clots are characteristically resistant to tissue plasminogen activator, and adjunctive DNase I administration restores clot susceptibility to lysis [161–164]. The bioresponsive co-delivery of tPA and DNase I in nanoparticle formulations dissolves tPA-resistant NET-rich clots within minutes in preclinical models [165], and microemulsion-based polysaccharide nanoparticle approaches expand the targeting modalities available for tissue-localized thrombolysis [166, 167]. The conceptual analogy to cancer is precise. NETs in the metastatic niche shield tumor cells from cytotoxic effectors, and NETs in the cerebrovascular clot shield the fibrin scaffold from lytic enzymes. The same DNase replacement strategy addresses both. The three preceding subsections have established that DNase replacement is therapeutic in autoimmunity, in ARDS, and in immunothrombosis, but the dose, route and selectivity that are optimal in each setting are quite different; the final subsection extracts these cross-disease lessons.

### Cross-disease lessons and the therapeutic window problem

The four non-tumor disease clusters discussed above expose a common challenge that did not arise in the cancer-only literature, namely the therapeutic window problem. Replacement of clearance enzymes, whether DNase or RNase, is therapeutic in cancer, SLE, COVID-19 ARDS, and immunothrombosis, but the dose, route, and selectivity that are optimal in each setting differ markedly. In cancer, intratumoral or peritumoral DNase delivery is desirable to maximize NET clearance and ICI synergy while limiting systemic effects on platelets and on chromatin in physiologic apoptosis. In SLE, systemic DNase activity must be elevated for sustained periods, but the immunogenicity of the recombinant enzyme, particularly DNASE1L3, which is itself an autoantigen, must be managed [124]. In acute ARDS or stroke, brief, high-dose DNase administration is preferred, with the goal of dissolving acute NET-rich clots, since chronic dosing is unnecessary and potentially counterproductive. The same logic operates in reverse for sensor inhibition. STING and cGAS inhibitors are therapeutic in chronic interferonopathies but would be expected to compromise antitumor immunity if administered systemically in a cancer-prone population [139, 140, 168–170]. Conversely, STING agonists are therapeutic in cancer but would be expected to exacerbate disease in pre-existing autoimmunity, and several preclinical autoimmune models confirm this risk. The therapeutic window therefore depends not only on the molecular target but on the disease context, dosing kinetics, and the patient's pre-existing immune tone.

A second cross-disease lesson concerns JAK inhibition. JAK1/2 blockade collapses the type I interferon signaling cascade downstream of both the DNA and RNA arms and is therapeutic in AGS and in interferon-high SLE [22, 146, 147]. The same biology, however, restricts antitumor IFN-driven immune surveillance, and the use of JAK inhibitors during active cancer therapy remains contraindicated outside narrow indications. The convergence on JAK as the most clinically mature downstream node across multiple interferonopathies illustrates that interventions distal to the sensor may achieve broader disease coverage at the cost of pathway specificity.

## Discussion

### Convergence: a two-fate framework for self DNA and RNA

Significantly, this review highlights that self DNA and self RNA typically follow divergent immunological trajectories. We posit that the DNase and RNase families act as a distributed regulatory network, with spatially segregated members playing a decisive role in determining whether self nucleic acids trigger immunity or remain silent. The biochemical and spatial heterogeneity of the family translates directly into distinct immunological outputs across the cytosolic, endolysosomal and extracellular niches. Although the framework above is anchored in tumor biology, the same compartmentalized clearance logic governs non-tumor pathology, where DNase insufficiency in systemic lupus erythematosus [150], TREX1 driven interferonopathy in Aicardi–Goutières syndrome [145], DNase responsive lung injury in COVID-19 associated ARDS [153], and NET-driven venous thromboembolism [156]. All manifest the same upstream failure mode that this review has brought into focus on the oncology side. Because a single upstream perturbation, sensing–clearance imbalance, generates phenotypically distinct diseases through a shared molecular grammar, the therapeutic entry points into these diseases are also unified, and it is the pharmacologic direction rather than the target itself that differs across settings.

### Open questions: cellular origin, the KIRC paradox, and sensor selectivity

Three inter-linked open questions remain unresolved and delimit the interpretive scope of this synthesis, and we treat them in the order in which they emerged from the mechanistic and translational chapters above rather than as an enumerated list. The most upstream unresolved question concerns the cellular origin of the nucleolytic activities we have attributed to specific compartments. For DNASE1L3, converging evidence identifies myeloid cells as the dominant source: circulating DNASE1L3 is produced by dendritic cells and macrophages, with macrophage-derived enzyme accounting for approximately 67% of total serum activity [34, 78], while the intratumoral pool originates from tumor-infiltrating dendritic cells, governing NET clearance and CD8⁺ T cell access [17]. This intratumoral DC-derived pool is distinct from the systemic hepatic pool [171], meaning plasma measurements may not reflect the nucleolytic capacity determining immune exclusion within tumors [172]. For DNASE2, both tumor cell–intrinsic and macrophage-extrinsic mechanisms operate: chromosomally unstable cancer cells upregulate lysosomal DNASE2 to suppress cGAS–STING activation [173–177], while tumor-associated macrophages independently elevate DNASE2 to manage phagocytic burden [178]. The extracellular DNASE1 deficit in advanced tumors, by contrast, reflects a multi-cellular failure in which neutrophil-derived DNASE1 is neutralized by stromal actin while cancer-associated fibroblasts promote NET deposition, creating a NET-permissive niche [179–182]. Because bulk transcriptomic data conflate these signals [183], resolving the causal hierarchy of DNASE dysfunction requires spatially resolved single-cell approaches that co-map nuclease expression, DNA debris, and immune cell positioning, a priority for future research in this field [184–188]. A parallel cellular origin question applies to NET driven disease outside the tumor microenvironment, where neutrophil derived NETs in COVID-19 lung injury [154], in cardiovascular and thrombotic disease [157], and in septic dysregulated immunity are governed by similar myeloid and endothelial deficits in DNASE1 and DNASE1L3 clearance capacity [155]

Downstream of the cellular-origin question sits a specific observation that the cellular-origin framework itself does not fully explain: the mechanistic basis for the DNASE1 paradox in clear-cell renal-cell carcinoma (KIRC), where elevated DNASE1 expression reported in published KIRC cohorts correlates paradoxically with adverse prognosis [189–192]. Three non-exclusive mechanisms are compatible with the current data: (i) tumor-infiltrating myeloid cells driving the observed expression signal; (ii) tumor-cell-autonomous DNASE1 expression that is enzymatically silenced by monomeric G-actin in the actin-rich KIRC microenvironment; or (iii) a mixture of both, and resolving these will require single-cell and spatially resolved KIRC transcriptomics together with matched G-actin concentration measurements. Equally unresolved is the question of why DNASE-derived DNA signals are preferentially channeled through cGAS rather than through AIM2 or TLR9. We propose this selectivity reflects a strict matching logic between the physicochemical properties of enzymatic products and the structural activation thresholds of distinct receptors: DNASE-mediated cleavage reduces long genomic DNA to intermediate-length double-stranded fragments that are too short to scaffold oligomeric AIM2 inflammasome assembly and too fragmented to preserve the contiguous unmethylated CpG motifs required for TLR9 recognition, while these cytosolic products are spatially excluded from the endosomal compartment where TLR9 resides [193–196]. This channeling model rests on fragment-length, structure–function, and compartment arguments derived from in vitro and murine studies and remains a working hypothesis, and direct comparative human evidence resolving the relative contribution of cGAS, AIM2 and TLR9 in specific tumor and autoimmune contexts is not yet available. We flag this explicitly throughout the review and treat the cGAS-preferential channeling as a candidate framework rather than an established mechanism, consistent with the broader evidentiary conservatism appropriate for a mechanistic proposal built on indirect data. The same channeling logic illuminates the AGS interferonopathy, where loss of TREX1, RNase H2, or SAMHD1 leaves cytosolic DNA and RNA:DNA hybrids unprocessed and funnels them onto cGAS–STING, producing the chronic type I interferon signature that defines the disease and that JAK1/2 blockade now partially controls in patients [21, 145, 197, 198].

### Caveats and the evidentiary frontier

Beyond these open questions, three interpretive limits bound the translational reach of the framework and dictate the caveats readers should carry forward. To make the evidentiary status of individual claims explicit, we adopt a three-tier system used elsewhere in the innate-immunity literature: Grade A denotes direct experimental evidence in human or in vivo systems, Grade B denotes biochemical or cell-line evidence with a clear mechanistic link, and Grade C denotes structural or biochemical inference awaiting direct test. The working hypotheses developed in this review—including the DNase-selectivity model that channels short cfDNA fragments preferentially through cGAS-STING relative to AIM2 and TLR9 (Grade C), the DNASE2-driven silencing model in cold tumors (Grade C), and the chronic-low-amplitude-STING-to-NF-κB switch (Grade C) are labelled at their point of introduction in the body text. The most operationally urgent of them is that the optimal therapeutic window for DNASE2 inhibition remains poorly defined: germline DNASE2 deficiency in mice produces lethal anemia and systemic interferon-driven pathology, raising concerns that pharmacologic inhibition could trigger autoimmune toxicity, and whether tumor-selective delivery systems such as mannose-receptor-targeted nanoparticles can achieve sufficient therapeutic index remains an open question [127, 199, 200]. Systemic administration of unguided DNases poses an analogous risk of disrupting physiological cell-free DNA homeostasis in healthy tissues [201], and next-generation therapeutics will need to be strictly coupled to precision delivery platforms, whether antibody-conjugated targeting of tumor-associated antigens [202], TME-responsive nanocarriers, or masked enzyme designs [203–207]. Sitting alongside this therapeutic-window problem is a distinct evidentiary limit: the current experimental foundation for several translational claims warrants scrutiny in its own right. Selective PAD4 inhibitors reduce NET-mediated tumor progression in preclinical models [208–212], yet randomized aerosol-dornase trials in COVID-19 ARDS have failed to demonstrate durable functional benefit [213, 214], underscoring that nucleolytic and citrullination-blocking agents developed in animal systems require rigorous human trial validation. The functional dissociation between DNASE1 transcript abundance and enzymatic activity in actin-rich microenvironments further shows that transcriptomic surrogates are insufficient proxies for nucleolytic function [215, 216]. The EDIM-Apo10 and fragmentomics-based diagnostic platforms have been validated primarily in single-center retrospective cohorts and require independent multi-institutional prospective replication before their sensitivity and specificity figures can be accepted as generalizable [217, 218]. The JAK1/2 inhibitor experience in AGS rests on multicenter retrospective series and single-arm prospective cohorts rather than placebo-controlled trials [21, 22], and the candidate biomarker layer combining interferon score, neurofilament light chain, and glycosphingolipid panels has not yet been harmonized across centers [148], so the durability and neuropathologic safety of long-term blockade still require prospective documentation [149]. Feeding directly out of both the therapeutic-window problem and the evidentiary limits above, a concrete set of research priorities emerges as the most forward-looking constraint that the field must now confront: high-throughput sequencing-based profiling of DNASE cleavage motifs across family members, atomic-resolution structural studies of DNASE2 with candidate active-site inhibitors to distinguish true competitive binders from promiscuous aggregators, single-cell spatial multi-omics of DNASE expression, DNA debris and cGAS–STING activation states across tumor evolution [219–221], engineered next-generation DNASE1L3 fusion proteins with extended half-life and enhanced chromatin-degrading capacity [124], and continued preclinical development of TREX1 inhibitors and ubiquitin-directed strategies targeting the cGAS-TREX1 axis [222, 223]. Beyond DNA-side modalities, the rapidly maturing ADAR1 inhibition and ZBP1 (Z-RNA/Z-DNA) biology represent adjacent axes that share platform chemistry with the DNase family [224–229]. Applied to non-tumor immunopathology, single-cell spatial profiling of NET-to-DNase clearance at sites of lupus nephritis, COVID-19 lung injury and atherothrombotic plaques is equally needed [230], and the bioresponsive NET-targeted nanocarriers being prototyped for cardiovascular, thrombotic and stroke indications share platform chemistry with the precision-delivery designs envisaged for tumor-selective DNase therapy [161, 166].

Looking ahead, several research priorities emerge with particular urgency. High-throughput sequencing-based profiling of DNASE cleavage motifs across family members is needed to establish the molecular grammar of nuclease-specific DNA fragmentation and enable rational design of fragmentomics classifiers. Atomic-resolution structural studies of DNASE2 in complex with candidate inhibitors are required to distinguish true competitive binders from promiscuous aggregators and guide structure-based medicinal chemistry toward clinically viable compounds. Complementing these molecular approaches, single-cell spatial multi-omics should be applied to map the temporal dynamics of DNASE expression, DNA debris accumulation, and cGAS–STING activation states across tumor evolution and in response to immunotherapy, providing the causal resolution that bulk analyses cannot achieve [219–221, 231]. The engineering of next-generation DNASE1L3 fusion proteins with extended half-life and enhanced chromatin-degrading capacity represents a tractable translational objective, given that preclinical evidence that DNASE1L3 restoration triggers PANoptosis and sensitizes tumors to anti-PD-1 therapy provides a compelling rationale for accelerated clinical development. Parallel preclinical programs in TREX1 inhibition have similarly advanced toward clinical readiness, with systemic TREX1 inhibitors and USP7-based TREX1 destabilizers demonstrating potent cGAS-STING activation and enhanced antitumor immunity in solid tumor models [222, 223], reinforcing the broader principle that selectively restoring or amplifying cytosolic DNA sensing represents a viable therapeutic axis distinct from extracellular NET clearance. Beyond DNA-side modalities, the rapidly maturing fields of ADAR1 inhibition and ZBP1 (Z-RNA/Z-DNA) biology represent adjacent therapeutic axes that share platform chemistry with the DNase family: ADAR1 inhibitors and nanovesicle-mediated ADAR1 silencing rewire interferon signaling and synergize with PD-L1 blockade [224, 225, 232], while ZBP1 activation triggers necroptosis and PANoptosis through Z-form nucleic acid sensing in cancer and antiviral immunity [226–229, 233], establishing a unified nucleic acid sensing pharmacology that bridges DNA clearance, RNA editing, and Z-form signaling. Taken together, the DNases family represents a mechanistically coherent and therapeutically actionable node within the DNA–immune axis of cancer biology, whose full translational potential will be realized only through the rigorous integration of structural, spatial, and clinical evidence. The same investigative agenda extends to non-tumor immunopathology. Single-cell spatial profiling of NET to DNase clearance at sites of lupus nephritis, COVID-19 lung injury, and atherothrombotic plaques is equally needed to resolve where clearance is overwhelmed [159], and the next generation of bioresponsive NET targeted nanocarriers being prototyped for cardiovascular, thrombotic, and ischemic stroke indications shares platform chemistry with the precision delivery designs envisaged for tumor selective DNase therapy [161, 165, 166], so insights and engineering investments are expected to flow in both directions between the two clinical contexts.

The scope of this review is deliberately focused on the six principal members of the mammalian DNase family (DNASE1, DNASE1L1, DNASE1L2, DNASE1L3, DNASE2, DNASE2B) and the core immune-relevant RNases (RNase L, RNase H1/H2, RNase T2). This boundary is principled rather than arbitrary: these enzymes share a common evolutionary origin traceable to two biochemically coherent superfamilies [6, 131], and operate as direct regulators of extracellular, circulating, cytosolic or lysosomal nucleic-acid pools that interface with innate immune sensing. Other nucleases with reported roles in cancer biology, including APEX1, FEN1 and the MRN complex, act primarily within DNA repair, replication fidelity or intracellular genome maintenance [234, 235], a mechanistic context fundamentally distinct from the DNA/RNA clearance-and-sensing interface that defines the enzymes covered here. It should nonetheless be acknowledged that this boundary is not absolute: TREX1 in particular shares functional overlap with DNASE1L3 in suppressing cGAS–STING activation through cytosolic DNA degradation [13, 14], and we have therefore included it explicitly across the mechanism and cancer-translation chapters. In a related vein that links the diagnostic platforms of this review to immune-checkpoint-inhibitor practice, recent multi-institutional cohorts have begun to validate ctDNA and cfDNA-derived fragmentomic and methylomic signatures as response biomarkers in ICI-treated solid tumors [236–239], indicating that the DNASE1L3-shaped cfDNA landscape developed here is poised to converge with the broader ctDNA-monitoring infrastructure for immunotherapy patient stratification. On the non-tumor side, coverage is centered on the four disease clusters where DNase/RNase biology has the most concrete clinical footprint; adjacent autoimmune entities including rheumatoid arthritis, systemic sclerosis and antiphospholipid syndrome share the NETosis-clearance axis but were not reviewed in depth, although the same DNase-replacement, NET-targeted-nanocarrier and OAS–RNase L pharmacology developed here will likely be relevant to their future translational evaluation [160, 162]. The synthesis therefore closes not with a claim of exhaustiveness but with an explicit acknowledgment of where the framework applies, where it is currently supported by direct evidence, and where the working hypotheses it identifies require targeted experimental confirmation.

## Conclusion

The DNase and RNase families together establish a structural and functional framework that links nucleic acid sensing, clearance, and immune signaling. Length specificity and subcellular localization of their catalytic products supply the molecular foundation for both diagnostic biomarkers and therapeutic intervention. In precision oncology this functional divergence supports dual track diagnostics through protein epitopes such as Apo10 and through fragmentomics of cell free DNA, while therapeutic translation depends on structure guided catalytic targeting, mannose receptor mediated lysosomal delivery, and single cell spatial validation of immune microenvironment remodeling. Beyond oncology the operational priority is reversed, with restoration of DNase and RNase activity becoming central to attenuating the chronic interferon signaling and NET driven tissue damage that underlie systemic autoimmune, monogenic interferonopathy, viral, and thrombotic disease. This bidirectional architecture converts mechanistic understanding of the DNA and RNA arms into a unified, clinically actionable framework across tumor and non-tumor settings.

## Data Availability

Not applicable.

## Abbreviations

ACC: Adrenocortical carcinoma
AUC: Area under the curve
cfDNA: Cell-free DNA
cGAMP: Cyclic GMP–AMP
cGAS: Cyclic GMP–AMP synthase
COAD: Colon adenocarcinoma
CRC: Colorectal cancer
DCs: Dendritic cells
DNase(s): Deoxyribonuclease(s)
dsDNA: Double-stranded DNA
EDIM: Epitope Detection in Monocytes
EMT: Epithelial–mesenchymal transition
ER: Endoplasmic reticulum
FDA: Food and Drug Administration
G-actin: Globular actin (monomeric actin)
GBM: Glioblastoma multiforme
GTEx: Genotype-Tissue Expression project
HCC: Hepatocellular carcinoma
HR: Hazard ratio
IAPs: Inhibitor of Apoptosis Proteins
Ki: Inhibition constant
KIRC: Kidney renal clear cell carcinoma
LIHC: Liver hepatocellular carcinoma
LUAD: Lung adenocarcinoma
MDSCs: Myeloid-derived suppressor cells
NETs: Neutrophil extracellular traps
NETosis: Neutrophil extracellular trap cell death
NK: Natural killer (cells)
OS: Overall survival
PAAD: Pancreatic adenocarcinoma
PANoptosis: Combined pyroptosis, apoptosis, and necroptosis
SARC: Sarcoma
TAMs: Tumor-associated macrophages
TME: Tumor microenvironment
Tregs: Regulatory T cells
